# *Bacillus cereus* PelA_DA_ is a polysaccharide de-*N*-acetylase required for pel-dependent biofilm formation

**DOI:** 10.1016/j.jbc.2026.113122

**Published:** 2026-05-07

**Authors:** Adithya S. Subramanian, Francois Le Mauff, Elena N. Kitova, Roland Pfoh, Mayura Panjalingam, Dung-Yeh Wu, Stephanie Gilbert, Zachary A. Morrison, Christian A. Jacobsen-Pérez, Erum Razvi, Mark Nitz, Jeroen Codée, John S. Klassen, Donald C. Sheppard, P. Lynne Howell

**Affiliations:** 1 Program in Molecular Medicine, The Hospital for Sick Children, Toronto, Ontario, Canada; 2 Department of Biochemistry, University of Toronto, Toronto, Ontario, Canada; 3 Infectious Disease and Immunity in Global Health Program, Research Institute of the McGill University Health Centre, Montreal, Quebec, Canada; 4 GlycoNet Integrated Services, Microbial Glycomic Node, Montreal, Quebec, Canada; 5 McGill Interdisciplinary Initiative in Infection and Immunity, Montreal, Quebec, Canada; 6 Department of Chemistry, University of Alberta, Edmonton, Alberta, Canada; 7 Institute of Chemistry, Leiden University, Leiden, Netherlands; 8 Department of Chemistry, University of Toronto, Toronto, Ontario, Canada; 9 Department of Microbiology and Immunology, Faculty of Medicine, McGill University, Montreal, Quebec, Canada

**Keywords:** *Bacillus cereus* ATCC 10987, biofilms, deacetylation, exopolysaccharides, mass spectrometry, Pel, structure-function, X-ray crystallography

## Abstract

Exopolysaccharides are key matrix determinants that provide structural integrity and regulate biomechanical properties of microbial biofilms. Biofilm exopolysaccharides often undergo modifications that determine their functional properties and localization. In *Bacillus cereus* ATCC 10987, PelA_DA_ expressed from the *pelDEA*_*DA*_*FG* operon is a putative deacetylase required for Pel-dependent biofilm formation. To understand the molecular basis of Pel deacetylation in *B. cereus* ATCC 10987, we determined the crystal structure of PelA_DA_ to 2.51 Å. PelA_DA_ adopts a distinct three-domain arrangement. We demonstrate *in vitro* that PelA_DA_ deacetylates α-1,4-linked GalNAc substrates in a length-dependent manner and that the N-terminal domain functions as a carbohydrate-binding module (CBM) capable of binding both GalNAc and partially deacetylated oligosaccharides. We found that the CBM domain together with the carbohydrate esterase (CE) domain forms an elongated carbohydrate binding cleft and that each domain is the founding member of two new CAZy families, CBM114 and CE26, respectively. Further, *in vivo* mutagenesis demonstrated that the catalytic activity of PelA_DA_ is required for Pel biosynthesis in *B. cereus* ATCC 10987. Employing AlphaFold, we propose a model wherein the N-terminal transmembrane helix of PelA_DA_ interacts with PelG. This interaction positions the protein to accept the polymer for deacetylation as it emerges from the cytoplasmic membrane. The work presented herein offers insight into the role of PelA_DA_ in Pel biosynthesis and modification in *B. cereus* ATCC 10987.

Biofilm formation is a common survival strategy employed by bacterial and fungal pathogens to protect themselves from environmental stressors, antibiotics, and the immune system (1, 2, 3). Biofilms are complex microbial communities encased in a hydrated self-produced extracellular matrix, composed of diverse biomolecules, including proteins, nucleic acids, and exopolysaccharides (4). Exopolysaccharides function as physical scaffolds facilitating nutrient and waste exchange and shape the three-dimensional architecture of biofilms (5, 6, 7).

The Pel exopolysaccharide was first identified in the matrix of the Gram-negative pathogen *Pseudomonas aeruginosa* (8, 9). In *P. aeruginosa*, Pel is a cationic polymer comprised of predominantly partially de-*N*-acetylated α-1,4-linked *N*-acetylgalactosamine (GalNAc Pel) (10). Pel performs multiple roles, including maintaining cell–cell interactions, modulating biomechanical properties of biofilms and enhancing resistance to aminoglycoside antibiotics (11, 12). Previously, using a novel bioinformatics pipeline, we identified *pel*-like operons in Gram-positive bacteria (13) and have shown in *Bacillus cereus* ATCC 10987 that each of the genes in the *pelDEA*_*DA*_*FG* operon is required for Pel biosynthesis and biofilm formation (14). Comparable to their roles in *P. aeruginosa*, PelF in *B. cereus* ATCC 10987 is a cytoplasmic glycosyltransferase which, when activated by the binding of c-di-GMP to the membrane protein PelD, polymerizes Pel from UDP-GalNAc. Pel is then predicted to be exported across the cytoplasmic membrane by PelG. PelD, PelF and PelG together with the membrane protein PelE, which is predicted to function as a protein-protein interaction hub, form the Pel biosynthetic complex (15). PelA_DA_ is a single-pass transmembrane protein that is predicted to be extracellular and have a carbohydrate esterase (CE) domain, suggesting that PelA_DA_ deacetylates the polymer to generate mature Pel. The genetic architecture of the Pel biosynthetic operon in *B. cereus* ATCC 10987 exhibits several differences from *P. aeruginosa*. A striking difference is that *B. cereus* PelA_DA_ is centrally located within the operon and is predicted to solely function as a polysaccharide deacetylase, while *P. aeruginosa* PelA has both deacetylase and glycoside hydrolase activity. In *B. cereus* ATCC 10987, a glycoside hydrolase (GH) domain is encoded in the divergently transcribed accessory gene *BCE_5582* (14, 16).

Deacetylation is a critical post-polymer modification that can affect the biosynthesis and localization of mature exopolysaccharides such as Pel, poly-β-1,6-*N*-acetylglucosamine (PNAG), and galactosaminogalactan (GAG) (17, 18, 19, 20). Deletion of exopolysaccharide deacetylase genes results in organism and strain-specific phenotypes. For example, in *P. aeruginosa* and *Yersinia pestis*, the deacetylases play a role in polymer synthesis as the lack of a functional enzyme leads to no detectable Pel or PNAG, respectively (17, 19, 21, 22). In certain Gram-positive PNAG-producing organisms, such as *S. epidermidis* and GAG-producing fungi such as *Aspergillus fumigatus*, deletion of their respective deacetylases leads to the secretion of fully acetylated non-adherent exopolysaccharide (23, 24). In *B. cereus* ATCC 10987, deletion of *pelA*_*DA*_ decreases biofilm adherence to levels comparable to a *ΔpelF* mutant, suggesting that modification of Pel by PelA_DA_ is critical for biofilm formation (14). While the presence of PelA_DA_ is required for biofilm formation, details of how it modifies the polymer at the molecular level are lacking.

Herein, we describe the structural and functional characterization of the *B. cereus* ATCC 10987 Pel polysaccharide deacetylase, *Bc*PelA_DA,_ which is the founding member of the CE26 family. The structure of PelA_DA_ reveals a tandem three-domain arrangement with an extended Pel binding groove formed by the central CE and N-terminal α/β/α-domains. PelA_DA_ deacetylates α-1,4-GalNAc oligosaccharides in a length-dependent manner. We demonstrate that the catalytic activity of PelA_DA_ is essential for the biosynthesis of Pel and biofilm formation in *B. cereus* ATCC 10987. Alphafold2 (AF2) and Alphafold3 (AF3) predict a transmembrane interaction between PelA_DA_ and PelG, providing a model for how Pel export across the cell membrane could be coupled to its chemical modification in Gram-positive bacteria.

## Results

### PelA_DA_ has a distinct three-domain structure

**Figure 1:**
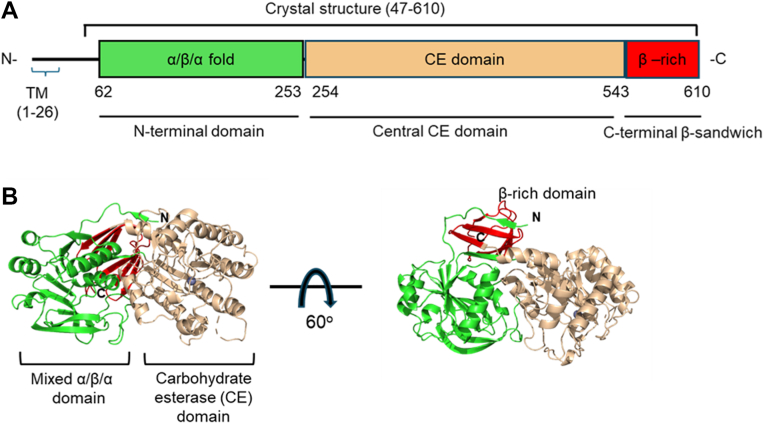
**PelA_DA_ is a three-domain protein required for Pel Biosynthesis.***A*, linear schematic of PelA_DA_ based on bioinformatic and structural analyses. *B*, cartoon representation of PelA_DA_. The N-terminal α/β/α, carbohydrate esterase (CE) and β-rich domains are colored in *green*, wheat, and *red*, respectively. N and C-termini of the protein are indicated.

Bioinformatic analyses suggest that *B. cereus* ATCC 10987 PelA_DA_ is a single-pass transmembrane protein with residues 27 to 610 located in the extracellular space. Residues 254 to 543 are predicted to have structural similarity to members of carbohydrate esterase (CE) family 4 (Fig. 1*A*). To gain insight into the structural and mechanistic features of PelA_DA_ and its role in the production of mature Pel, we determined the crystal structure of PelA_DA_ lacking its predicted transmembrane region (PelA_DA_^27-610^) to 2.51 Å resolution using molecular replacement (Table 1). Two molecules of PelA_DA_^27-610^ (chain A and chain B) are present in the asymmetric unit. The quality of the electron density allowed us to model residues 47 to 269, 278 to 610 in chain A, and 47 to 269, 281 to 608 in chain B. Both molecules exhibit a high degree of similarity to each other with a root-mean-square deviation of 0.3 Å over 3481 atoms between the proteins (Fig. S1). PelA_DA_^27-610^ has a three-domain arrangement (Fig. 1*B*). Residues 47 to 62 form a loop that leads into the N-terminal domain. The N-terminal domain, residues 62 to 253, adopts a mixed α/β/α fold comprised of a nine-stranded β-sheet core surrounded by four α-helices. Residues 254 to 543 adopt a distorted (β/α)_7_ fold, reminiscent of the topology found in other carbohydrate esterases. The C-terminal domain features a core six-stranded β-sandwich that is augmented by an additional β-sheet (residues 248–253) that bridges the N-terminal α/β/α and CE domains. In addition, the N-terminal region (residues 47–62) caps the top of this β-sandwich domain (Fig. 1*B*).

**Table 1:** X-ray data and refinement statistics

	PelA_DA_^27-610^
Data collection	
Wavelength (Å)	1.28377
Temperature (K)	100
Space group	*P* 1 2_1_1
Cell dimensions	
*a, b, c* (Å)	55.2, 111.9, 100.4
*α, β, γ* (°)	90.0, 90.8, 90.0
Resolution (Å)	29.64–2.51 (2.58–2.51)^a^
Total No. reflections	139,988 (9108)
No. of Unique Reflections	40,986 (2720)
R_merge_ (%)^b^	8.9 (60.00)
Average I/sigma (I)	7.8 (1.9)
Completeness (%)	98.5 (89.8)
Multiplicity	3.4 (3.3)
CC1/2	0.997 (0791)
Refinement	
Resolution (Å)	29.64–2.51
R_work_/R_free_ (%)^c^	19.41/25.63
No. atoms	
Protein	8751
Zn^2+^	2
Solvent	77
B-factors	
Macromolecules	66.49
Zn^2+^	68.47
Solvent	53.69
R.m.s. deviations	
Bond lengths (Å)	0.008
Bond angles (°)	1.00
Ramachandran plot^d^	
Total favored (%)	96.24
Total allowed (%)	3.76
PDB	9ZAU

a The values in parentheses correspond to the highest resolution shell.

b $R_{\text{merge}}=\sum \sum |I\left(k\right)\;–\;|/\sum I\left(k\right)$, where I(k) and I represent the diffraction intensity values of the individual measurements and the corresponding mean values, respectively. The summation is over all unique measurements.

c $R_{\text{work}}=\sum \left|\left|F_{\text{obs}}\right|–\;k\right|F_{\text{calc}}\left|\left|/\right|F_{\text{obs}}\right|$, where F_obs_ and F_calc_ are the observed and calculated structure factors, respectively. R_free_ is the sum extended over a subset of reflections (10%) excluded from all stages of the refinement.

d As calculated using PHENIX.

The molecular interface between chain A and chain B is primarily facilitated by residues in the loop regions of the CE and α/β/α domains (Fig. S1*B*). The two chains are positioned such that the CE domain of chain A packs against the N-terminal domain of chain B and *vice versa*, thus resulting in a head-to-toe orientation of the two proteins. This interface is unlikely to be biologically relevant, as results from the Proteins, Interface, Surfaces, and Assemblies (PISA) (25) server indicate a complexation significance score of 0.0, and size exclusion chromatography of PelA_DA_^27-610^ suggests that the protein is a monomer in solution.

Examination of the PelA_DA_ difference electron density map revealed large 17 and 15σ peaks in the active site groove of the CE domain of chain A and B, respectively, suggesting that PelA_DA_ binds metal ions. Given that we had soaked the crystals prior to data collection in 200 mM ZnSO_4_ to use for phase determination, we assume this peak represents Zn^2+^. Structural analysis revealed a distorted trigonal bipyramidal geometry with the zinc atom coordinated by D263, H350, H354, H488 and one water molecule in chain A. In chain B, there is a 2.5 Å shift in the rotamer conformation of the H488 side chain compared to chain A, and instead, a second water molecule is involved in coordinating the Zn^2+^ ion (Fig. S2).

### The CE domain of PelA_DA_ shares structural features with CE4, CE18, and CE21 enzymes

To assess the functional roles of PelA_DA,_ we began by studying each domain individually. To determine if the CE domain of PelA_DA_ can be classified into an existing family, we submitted the primary structure to the dbCAN3 automated carbohydrate annotation server (26). The Hidden Markov Model profile of PelA_DA_ has low overlap with the CE families annotated to date. This analysis suggested that if PelA_DA_ was enzymatically active, it could be the founding member of a new CE family. We next sought to identify homologs of PelA_DA_ to provide insight into its function and identify whether the protein contained the key CE motifs required for enzymatic activity (27). Searches using the DALI server suggested that the CE domain of PelA_DA_ shares structural similarities with CE4 and CE18 enzymes (Fig. S3) (27). Among the top hits that have been functionally characterized were the CE18 GAG deacetylase Agd3 (PDB: 6NWZ, sequence identity of 18.98%, RMSD of 3.90 Å over 897 atoms) and the CE4 peptidoglycan deacetylase PgdA (PDB: 2C1G, sequence identity of 23.67%, RMSD of 4.71 Å over 861 atoms) (20, 28). Given the low sequence identity with Agd3 and PgdA we expanded our structural and bioinformatic analysis to include two additional well-characterized CE4 Gram-positive polysaccharide deacetylases listed among the top 100 DALI hits: the PNAG deacetylase IcaB from *Ammonifex degensii* (PDB: 4WCJ, sequence identity of 24.47%, RMSD of 2.82 Å over 646 atoms) and the canonical CE4 enzyme Bc1960 from *B. cereus* ATCC 14579 (PDB: 4L1G, sequence identity of 15.26%, RMSD of 4.59 Å over 854 atoms) (29, 30) as well as the AF2 model (residues 521–804) of the CE21 Pel deacetylase from *P. aeruginosa* *Pa*PelA (sequence identity of 19.6%, RMSD of 3.95 Å over 961 atoms) (31). The *Pa*PelA AF2 model is very similar to the recently determined crystal structure of *Pseudomonas thermotolerans* PelA (1.6 Å RMSD over 816 α-carbons) (31). As *Pt*PelA lacked density for several important loops, we have used the AF2 model for our comparisons.

**Figure 2:**
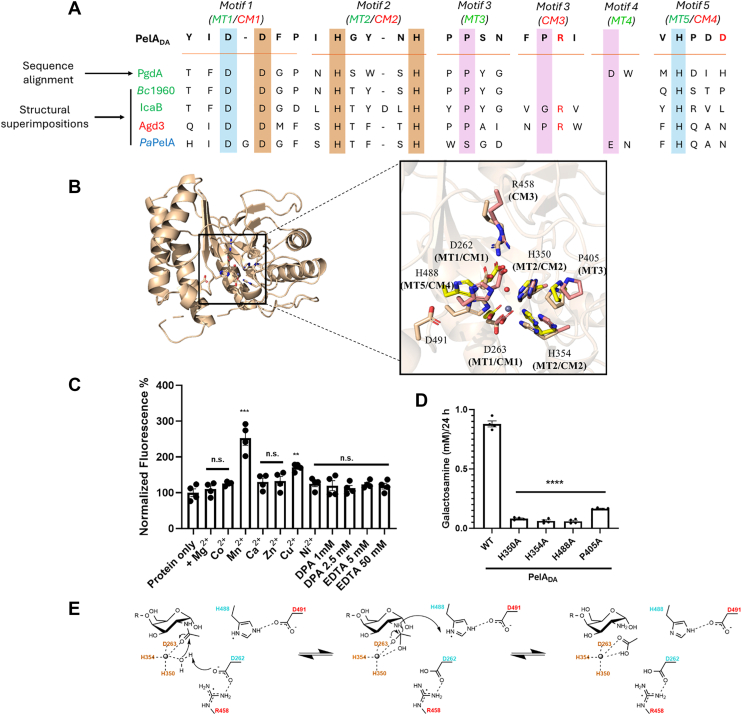
**PelA_DA_ is a metalloenzyme that shares homology to CE4, CE18 and CE21 enzymes.***A*, sequence alignments of PelA_DA_ with CE4 (PgdA, *Bc* 1960, IcaB, *green*), CE18 (Agd3, *red*) and CE21 (*Pa*PelA *dark blue*) family members displaying catalytic motifs (MT1-5/CM1-4). Catalytic residues were identified using Clustal Omega sequence alignments with PgdA and structural superimpositions with Bc1960, IcaB, Agd3 and *Pa*PelA. The putative catalytic base (D262) and acid (H488) are highlighted in *light blue*. Residues involved in Zn^2+^ coordination are highlighted in *brown*. Other key residues involved in substrate binding or catalysis are highlighted in *light magenta* or depicted as *bold red letters*. *B*, cartoon representation of the CE domain of PelA_DA_ (chain A) along with a structural alignment of catalytic residues Agd3 (6NWZ, *pink*) and PgdA (2C1G, *yellow*). The Zn^2+^ ion and water molecules found in PelA_DA_ are displayed as *grey* and *red spheres*, respectively. *C*, AMMU assay testing the esterase activity of PelA_DA_ in the absence or presence of indicated divalent cations and metal chelators. Error bars represent the standard error of the mean of two independent experiments (n = 4). A significant difference is indicated (∗∗, *p* < 0.01, ∗∗∗, *p* < 0.001) as determined by unpaired *t* tests between the protein only group and respective metal or chelator additions. *D*, fluorescamine assay probing the deacetylase activity of PelA_DA_ wild-type and catalytic mutants using synthetic α-1,4-(GalNAc)_9_. Error bars represent the standard error of the mean of two independent experiments (n = 4). A significant difference is indicated (∗∗∗∗, *p* < 0.0001) as determined by unpaired *t* tests between the wild-type and mutants. *E*, proposed de-*N*-acetylation reaction for PelA_DA_. The residues are coloured as in panel A. AMMU, acetoxymethyl-4-umbelliferone; DPA, dipicolinic acid; EDTA, ethylenediaminetetraacetic acid.

In CE4/CE21 and CE18 family enzymes, the key residues required for catalysis are found in distinct motifs, designated as MT1 to MT5 (32) and CM1 to CM4 (20), respectively. The key catalytic residues of MT1, MT2, and MT5 in PelA_DA_ were identified by sequence and structural alignment with PgdA (Fig. 2, *A* and *B*). The predicted catalytic base/acid pair D262 and H488 were identified as part of MT1/CM1 and MT5/CM4 and are located at the ends of β1 and β7, respectively. Two conserved histidine residues were identified as part of the “HxxxH” MT2/CM2 motif which with D263 in MT1/CM1 coordinate the Zn^2+^ atom. We also identified a conserved proline residue (P405) which is typically found in MT3 in CE4 enzymes. In contrast, the subsequent motifs identified in the primary structure of PelA_DA_ resemble that of CE18 enzymes. Specifically, two key similarities were identified: the first being the presence of a base coordinating arginine (R458) located downstream of the proline in MT3 which forms a bidentate salt bridge with D262. The second similarity is the absence of a conserved aspartic acid located in MT4 that is involved in activating the catalytic acid. In Agd3, an ordered water molecule activates the catalytic MT5/CM4 histidine; however, an equivalent water molecule is not present in the PelA_DA_ structure. Instead, an aspartic acid (D491) that immediately follows MT5 is positioned to activate H488. Due to the structural differences described, it appears that PelA_DA_ might adopt a distinct, slightly altered catalytic mechanism in comparison to traditional CE4/CE18/CE21 enzymes.

In addition to variations in the primary structure between PelA_DA_ and other CE enzymes, we identified topological differences that shape the active site architecture of PelA_DA_ (Fig. S4*A*). The loops connecting β_1_/α_1_ (L1) and β_2_/α_2_ (L2) in PelA_DA_ create a longer groove on the top face of the β/α-fold of PelA_DA_ than seen in PgdA (Fig. S4, *A* and *D*) (28). Additionally, loops L3 and L4, which connect β3-α3 and β5-β6, respectively, form one face of the active site groove of PelA_DA_. The orientation of loops L3 and L4 position them perpendicular to the active site cleft, creating a deeper polymer-binding groove compared to PgdA (Fig. S4, *A* and *D*). In contrast, Agd3 has a wider and longer active site cleft than PelA_DA_ (Fig. S4, *B* and *D*). The L2 loop in Agd3 (termed the β2 insertion) is significantly longer and creates an electronegative extension on one face of the active site groove of Agd3. Loop L3 in PelA_DA_ partly overlaps the β2 insertion of Agd3 in structural superimpositions. However, the positioning of L3 along the active site groove and loop L5 connecting β6-α5 in PelA_DA_ narrows the active site cleft to 11.7 Å (Fig. S4, *B*, *D* and *E*). Key structural divergences also occur between PelA_DA_ and the AF2 model of the deacetylase domain of *Pa*PelA. *Pa*PelA harbours a unique extended loop containing α5, with the terminal part of this loop partially overlapping L4 of PelA_DA_. L3 of PelA_DA_ is replaced by a longer α/α insertion in *Pa*PelA that forms a deeper active site cleft compared to PelA_DA_ (Fig. S4, *C* and *D*). Taken together, our structural superimpositions suggest a distinct binding cleft in the CE domain of PelA_DA_ that is different from other CE family enzymes.

### PelA_DA_ is a metal-dependent enzyme that deacetylates synthetic GalNAc oligosaccharides

Previously, work characterizing CE4, C18 and CE21 enzymes has demonstrated the importance of a conserved Asp-His-His triad in coordinating an active site metal ion (20, 29). CE enzymes often display a preference for binding divalent cations such as Zn^2+^ and exogenous addition of metals can increase the activity of CE enzymes *in vitro* (33, 34). To test if PelA_DA_ is an active CE enzyme *in vitro* and understand its metal preferences, we used the pseudosubstrate acetoxymethyl-4-methylumbelliferone (AMMU) to test for esterase activity (Fig. 2*C*) (35, 36). As isolated, wild-type PelA_DA_ displayed ∼2.5 and 1.7 fold higher activity after incubation with Mn^2+^ and Cu^2+^, respectively, compared to when no exogenous metal was added (Fig. 2*C*). As seen previously for *Escherichia coli* PgaB, PelA_DA_ activity was not abolished upon the addition of chelators such as EDTA and DPA (37). The inability of chelators to reduce PelA_DA_ esterase activity suggests that the bound divalent cation is not kinetically labile and is tightly coordinated by residues in MT1, MT2 and MT5 and shielded from chelation. Our results indicate that, akin to other characterized CE4, CE18, and CE21 enzymes, a divalent cation plays a role in the catalytic activity of PelA_DA._

Using AMMU as a pseudosubstrate only enables us to probe the initial step of the deacetylation reaction. Therefore, to assess deacetylase activity and the role of the identified catalytic motifs in the enzymatic mechanism of PelA_DA_, we used a chemically synthesized nonamer of α-1,4-GalNAc as a substrate. Wild-type PelA_DA_ and eight active site mutants were recombinantly expressed and purified (Fig. S5, Table S1). We found there was aggregation and significant degradation of the D262N, D263N, D490N, and D491N point mutants, precluding their use in our assays (Fig. S5). The stability of the H350A, H354A, H488A and P405A mutants was assessed using circular dichroism spectroscopy and their melting temperatures were found to be similar to that of the wild-type protein (Table S1). To assess the deacetylase activity of PelA_DA_ and the four stable mutants, we used a fluorescamine assay, which detects the primary amines generated as a product of the deacetylation reaction (38). The fluorescamine assay results for wild-type PelA_DA_ show an increase in free amine concentration after 24 h (Fig. 2*D*). Mutation of the metal-coordinating residues H350, H354, and the catalytic acid H488 resulted in no detectable free amines. While free amines could be detected for the MT3 P405A mutant, the levels detected were significantly reduced (∼5-fold) relative to wild-type PelA_DA_ (Fig. 2*D*). Combined, our results suggest that PelA_DA_ is an active polysaccharide deacetylase and residues involved in coordinating the metal are required for activity.

### PelA_DA_ displays length-dependent deacetylation of α-1,4-GalNAc oligosaccharides

**Figure 3:**
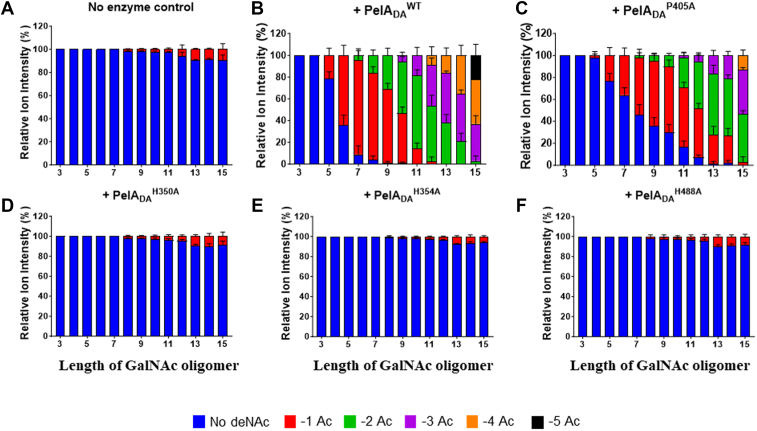
**PelA_DA_ differentially deacetylates GalNAc oligomers of various lengths.** MALDI-TOF MS analysis of α-1,4-(GalNAc) oligomers isolated from the biofilm matrix of *A. fumigatus* and treated with the glycoside hydrolase Sph3. Relative intensity of deacetylation products in isolation (*A*) and after incubation with PelA_DA_ wild-type (*B*) or indicated catalytic mutants (*C–F*) for 24 h. Ac, acetylation; DeNAc, de-*N*-acetylation.

Having demonstrated *in vitro* activity of PelA_DA,_ we next sought to directly analyze the products of the deacetylase reaction to investigate the pattern of deacetylation (10). To determine if there exists a correlation between the number of deacetylation events and substrate length, a mixture of α-1,4-GalNAc oligosaccharides ranging from 3-mers to 15-mers were incubated with either wild-type PelA_DA_ or the mutants for 24 h, and the products were then analyzed using matrix-assisted laser desorption/ionization time-of-flight mass spectrometry (MALDI-TOF MS) (20, 39) (Figs. 3 and S6). Even in the absence of PelA_DA_, we observed that oligosaccharides longer than 7-mers had a basal level of deacetylation, with up to 10% of the 13 to 15-mers exhibiting a maximum of one deacetylation event (Fig. 3*A*). In the presence of wild-type PelA_DA_, the shortest oligosaccharide to be deacetylated were the 5-mers, where 20% were mono-deacetylated (Fig. 3*B*). We observed that the number of deacetylation events correlated with the length of oligosaccharides with larger polymers being deacetylated multiple times (Fig. 3*B*) (20, 40). The nonamer was the first oligomer where we could no longer detect appreciable amounts of non-deacetylated product. The longest oligosaccharides used in our assay were 15-mers. These oligomers were deacetylated multiple times, with ∼22% of the 15-mers undergoing five deacetylation events (Fig. 3*B*). Our fluorescamine assay had revealed that the P405A mutant retained residual esterase activity. In keeping with this result, we found that <3% of the 5-mer population was deacetylated after the 24-h incubation period, while ∼24% of the 6-mer population had at least one deacetylation event. As for the wild-type enzyme, a positive correlation between the number of deacetylation events and oligosaccharide size was observed for the P405A mutant. In keeping with the reduced activity of this mutant we found that the 13-mer was the first oligomer that was completely processed and only four deacetylation events were observed in <13% of the 15-mer population (Fig. 3*C*). Additionally, comparable to our fluorescamine assay results, incubation of the oligosaccharide mixture with the H350A, H354A and H488A mutants revealed only basal levels of deacetylation, similar to our no enzyme control, further reinforcing the importance of these residues for catalysis (Fig. 3, *D–F*). Combined, these data suggest that the minimal substrate length required for deacetylation by wild-type PelA_DA_ is a 5-mer. The P405A mutant retains deacetylase activity, although fewer deacetylation events are observed compared to the WT enzyme as the length of the oligomer increases.

To further understand how PelA_DA_ processes the Pel polymer, an MS-MS fragmentation study of the mono-deacetylated 8-mer produced after incubation with either wild-type PelA_DA_ or P405A was performed (Fig. S7, Table S3). This analysis was unable to conclusively determine the location of the de-*N*-acetylation event. We found that fragmentation of the first two residues at the reducing and non-reducing ends of the oligomer was consistent with the presence of only HexNAc. In comparison, the ions generated by fragmentation within the central region of the oligosaccharides, that is, sugar residues 3 to 6, seem to originate from different isomers of mono-de-*N*-acetylated GalNAc_8_. Interestingly, no differences could be observed in the fragmentation patterns of the oligosaccharide incubated with the wild-type or P405A enzyme (Fig. S7, Table S3). Although our MS-MS analysis failed to conclusively deduce the pattern of mono-deacetylation for GalNAc_8_, it does suggest that the central region of the oligosaccharide is more susceptible to modification by either the wild-type or the P405A mutant. Our analysis of how the different length oligosaccharides are deacetylated by the P405A mutant is also consistent with the reduced catalytic activity observed in our fluorescamine assay (Fig. 2*D*).

### The catalytic activity of PelA_DA_ is required for biofilm formation and Pel biosynthesis

**Figure 4:**
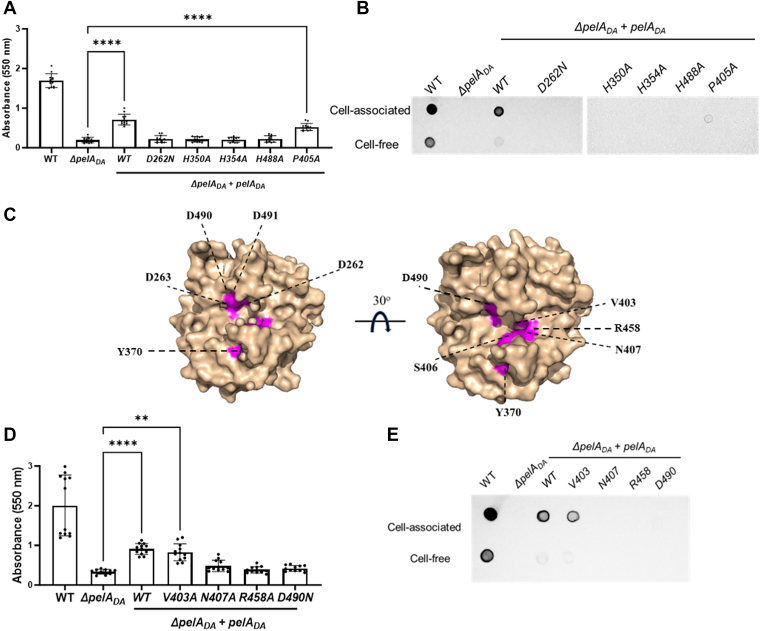
**Pel biosynthesis requires enzymatically active PelA_DA_.***A* and *D*, surface adherence of the wild-type and indicated catalytic or substrate binding mutants was assayed using crystal violet staining of 24 h biofilms. Error bars represent the standard error of the mean of two independent experiments (n = 12). Statistics were calculated using one-way ANOVA with a Dunnett’s multiple comparison test (∗∗∗∗, *p* < 0.0001, ∗∗, *p* < 0.01). *B* and *E*, *dot* blot analyses of cell-associated and cell-free fractions from *B. cereus* ATCC 10987 strains using an anti-(GalNAc)_3_ monoclonal antibody to detect Pel production. *C*, cartoon representation of the CE domain of PelA_DA_ (PelA_DA_^254-528^) depicting the catalytic and putative substrate binding residues targeted for mutagenesis.

Previous work has demonstrated that transposon insertions in *pelA*_*DA*_ and a non-polar deletion of the gene result in the loss of biofilm formation (14, 41). We next wanted to establish whether the biofilm phenotype was dependent on the catalytic activity of PelA_DA_ or simply the presence of the protein. To test this hypothesis, *pelA*_*DA*_ wild-type and point mutants of the catalytic domain were generated using the pAD123-P_xyl_ vector and complemented *in trans* into the *ΔpelA*_*DA*_ strain. As expected, adherence to biofilm plates was abrogated in the *ΔpelA*_*DA*_ strain (Fig. 4*A*). Adherence was restored by complementation with wild-type *pelA*_*DA*_ (*ΔpelA*_*DA*_*+pelA*_*DA*_) but not with MT1/2 catalytic mutants (Fig. 4*A*). In keeping with our enzyme assays (Figs. 2*D* and 3), complementation with the P405A mutant also restored adherence (Fig. 4*A*). To ensure that the phenotype observed for the MT1/2 mutants was not due to the lack of *pelA*_*DA*_ expression, Western blotting with a polyclonal PelA_DA_ antibody was performed (Fig. S8). Expression of PelA_DA_ in the parental *B. cereus* ATCC 10987 strain was not detected due to the low basal level of expression under the growth conditions tested. However, all catalytic mutants were expressed to similar levels as the wild-type complementation strain. These results suggest that biofilm formation in *B. cereus* ATCC 10987 requires the presence of enzymatically active PelA_DA_.

Carbohydrate esterases have diverse roles in modification, secretion, and the production of the cell-associated and cell-free forms of exopolysaccharides (33, 42). We hypothesized that the decrease in biofilm adherence observed for the *ΔpelA*_*DA*_ and the MT1/2 complementation mutants was due to changes in the levels of Pel. We used dot blot analyses to probe for Pel in the cell-associated and cell-free fractions of the wild-type and mutant *B. cereus* strains using a monoclonal antibody raised against (GalNAc)_3_ (43). A robust signal was detected for both cell-associated and cell-free fractions in the wild-type strain (Fig. 4*B*). In contrast, no signal for either fraction was detected in the *ΔpelA*_*DA*_ mutant. The signal for cell-associated and cell-free Pel was restored in the *ΔpelA*_*DA*_ + *pelA*_*DA*_ strain, although the signal for cell-free Pel is significantly less than in the wild-type strain (Fig. 4*B*). The signals for both cell-free and cell-associated Pel in the wild-type complemented strain is in keeping with our crystal violet assay, where the level of adherence measured was ∼one-third that of the wild-type strain. Similar to our crystal violet assay results, complementation with the catalytically inactive *pelA*_*DA*_ MT1 and MT2 mutants resulted in a phenotype similar to the *ΔpelA* deletion, with no Pel detected in either cell-free or cell-associated fractions. Of note, the *ΔpelA*_*DA*_ + *pelA*_*DA*_^*P405A*^ strain did produce a weak yet detectable signal in the cell-associated fraction (Fig. 4*B*). Qualitatively, our dot blot assay results, like the crystal violet assay, suggest that the enzymatic activity of PelA_DA_ is required for the biosynthesis of cell-associated and cell-free Pel in *B. cereus* ATCC 10987.

Further examination of the PelA_DA_ substrate-binding groove allowed us to identify conserved residues within 10 Å of the bound Zn^2+^ ion that might play a role in substrate binding and/or be important for activity (Fig. 4*C*). We selected to mutagenize five putative substrate-binding residues and R458, predicted to coordinate the catalytic base D262, and study their contributions to PelA_DA_-dependent biofilm formation and Pel biosynthesis. No detectable protein levels were observed on a Western blot when residues Y370 and S406 were mutated; hence, these were not studied further (Fig. S8). Reduced protein production was observed when Δ*pelA*_*DA*_ was complemented with the R458A mutant compared to the wild-type protein (Fig. S8). Complementation with the V403A mutant resulted in a statistically significant increase in adherence levels compared to the *ΔpelA*_*DA*_ strain and a signal for Pel in both the cell-associated and cell-free Pel fractions, indicating that this residue is dispensable for PelA_DA_ activity (Fig. 4, *D* and *E*). Conversely, mutations in residues N407, R458, and D490 resulted in adherence-negative phenotypes with no Pel detected in either fraction (Fig. 4, *D* and *E*). Our data suggest that residues N407 and D490 contribute to PelA_DA_-dependent biofilm formation likely through substrate binding, while R458 plays an important role in the catalytic mechanism of the enzyme.

### The N-terminal α/β/α domain of PelA_DA_ functions as a carbohydrate-binding module (CBM)

**Figure 5:**
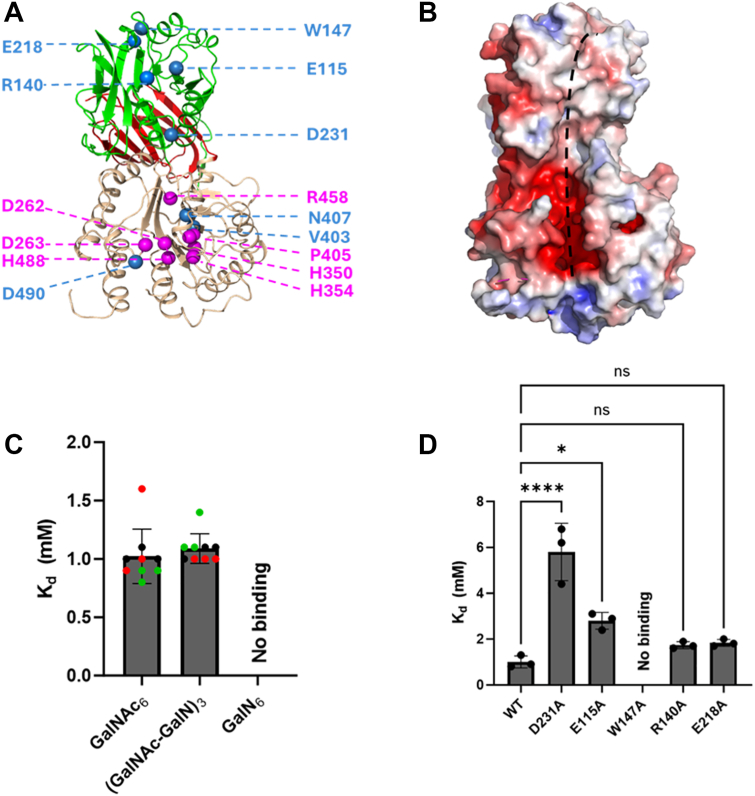
**The N-terminal α/β/α domain of PelA_DA_ functions as a carbohydrate binding module (CBM).***A*, Cartoon representation of PelA_DA_ showing the CBM in *green*, CE domain in wheat and β-rich domain in *red*. Catalytic residues and predicted substrate binding residues are depicted as *magenta* and *blue spheres*, respectively. The first conserved aspartic acid residue (D231) of the CBM is ∼27 Å from the catalytic base (D262). *B*, electrostatics surface representation showing that the shallow cleft of the α/β/α domain extends from the predominantly electronegative active site cleft of the CE domain. Electrostatics were calculated by APBS in PyMol (v. 2.4) and visualized in *blue* to *red* (+5 kT/e to −5 kT/e). *C*, dissociation constants (*K*_*d*_) for PelA_DA_^27-253^ for α-1,4-linked GalNAc oligosaccharides as determined by ESI-MS. For (GalNAc)_6_, measurements were performed at 100 μM (*black*), 200 μM (*green*), and 300 μM (*red*); for the (GalNAc-GalN)_3_, measurements were performed at 100 μM (*black*), 120 μM (*green*), and 140 μM (*red*). Each data point represents results from an independent ESI-MS measurement at the indicated concentration. *D*, dissociation constants (*K*_d_) for PelA_DA_^27-253^ wild-type and CBM mutants for α-1,4-(GalNAc)_6_ as determined by ESI-MS. Error bars represent the standard error of the mean of three independent experiments (n = 3). Statistics were calculated using one-way ANOVA with a Dunnett’s multiple comparison test (∗, *p* < 0.05, ∗∗∗∗, *p* < 0.0001).

Our MALDI-TOF MS analyses demonstrate that PelA_DA_ can bind and deacetylate longer oligosaccharides more efficiently than shorter ones (Fig. 3). Examination of the structure of PelA_DA_ reveals that the neutral cleft of the N-terminal α/β/α domain is oriented in tandem with the CE domain. Given that we anticipate that the CE domain possesses multiple sugar binding sites, we hypothesized that the α/β/α domain could facilitate binding of longer Pel polymers (Fig. 5*B*). This hypothesis is supported by our structural similarity searches using DALI, which suggest that the N-terminal α/β/α domain of PelA_DA_ shares structural similarity with the α/β/α domain of the fungal deacetylase Adg3 (PDB: PDB: 6NWZ, RMSD of 2.5 Å over 642 atoms) (20). The N-terminal domain α/β/α of Agd3 is required for substrate binding and increases the stability of the CE domain. Structural superpositions of the α/β/α domains of PelA_DA_ and Agd3 identified several topological differences (Fig. S9*A*). The most striking difference is that while the α/β/α domain of Agd3 contains a cleft with several conserved aromatic and acidic residues that facilitate substrate binding, this region in PelA_DA_ is replaced by two β-sheets that cap the top of the domain (Fig. S9*A*). We aligned the α/β/α domains of Agd3 and PelA_DA_ to further differentiate the architectures. The resulting structural alignments indicate a shift in the relative positioning of the CE domains of PelA_DA_ and Agd3 (Fig. S9*B*). The binding groove between the CE and α/β/α domains of Agd3 adopts a prominent linear arrangement. In contrast, the shift in the CE domain of PelA_DA_ creates a curved binding groove with partially conserved charged and aromatic residues (Figs. 5, *A* and *B*, and S8*B*). To determine whether the α/β/α domain of PelA_DA_ acts as a carbohydrate binding module, provides an extended polymer binding surface and the specificity of the oligosaccharides it binds, we recombinantly expressed and purified the isolated α/β/α domain (PelA_DA_^27-253^). Next, we tested whether chemically synthesized fully acetylated, partially deacetylated, and fully deacetylated GalNAc hexamers bound to the protein using electrospray ionization mass spectrometry (ESI-MS). ESI-MS detected a single species for PelA_DA_^27-253^ with a molecular weight of 26,693.5 ± 0.7 Da, which agrees with the expected mass of 26,693.5 Da. PelA_DA_^27-253^ bound (GalNAc)_6_ and (GalNAc-GalN)_3_ with *K*_d_’s of 1.1 ± 0.2 mM and 1.1 ± 0.1 mM, respectively, while no binding was observed with (GalN)_6_ (Figs. 5*C* and S10). The ability of PelA_DA_^27-253^ to bind fully acetylated and partially deacetylated oligomers is consistent with surface electrostatics that indicate a slightly electronegative surface along the binding groove. These results confirm that PelA_DA_^27-253^ functions as a carbohydrate-binding module (CBM) and displays preferential binding for fully acetylated or partially deacetylated α-1,4-GalNAc oligomers over fully deacetylated oligomers. As, PelA_DA_^27-253^ could not be assigned to any known CBM families due to its low sequence identity to characterized CBM domains, this domain is the founding member of a new CBM family, CBM114.

Polysaccharide binding sites in CBMs often involve surface-exposed aromatic and charged residues. Using our structure and comparison with the CBM of Agd3, a member of the CBM 87 superfamily, we identified four residues in the binding cleft that we hypothesized are involved in polymer binding. Residue D231 is located at the base of the CBM proximal to the CE domain and is equivalent to the first conserved residue W314 in the CBM of Agd3 (Fig. 5*A*). Consurf analysis revealed that R140 and E115 are highly conserved, while W147, which is located towards the terminal end of the CBM binding groove is less conserved (Fig. 5*A*). To further probe the role of these residues, we generated alanine mutants and recombinantly expressed, purified, and characterized their ability to bind (GalNAc)_6_. As a control we used an E218A mutant. E218 is located on the opposite face of the CBM binding cleft and therefore not expected to affect the *K*_d_ of binding. When assayed for their ability to bind α-1,4-(GalNAc)_6_, we noticed a ∼5-fold and 3-fold increase in the *K*_d_ of the D231A and E115A mutants, respectively. No detectable binding was observed for the W147A mutant (Figs. 5*D* and S11). Conversely, no statistical difference in binding was observed between the wild-type, R140A mutant and the E218A control. Taken together, our results suggest that residues D231, E115 and W147 aid in alignment of oligosaccharides by facilitating substrate binding in the CBM of PelA_DA_.

To further evaluate the CBM binding groove, we employed AF3 to predict the binding modes for (GalNAc)_6_ to the PelA_DA_^27-253^ (Fig. S12). Conformational sampling confirmed that the oligosaccharide bound to the groove we had identified from our structural and mutagenesis studies. CBM-(GalNAc)_6_ interactions analyzed by LIGPLOT (44) indicate that the second, third and sixth sugar units of the hexamer are stabilized by E115 and D231 (Fig. S12*C*). The side chain of E115 makes van der Waals contacts with the C3-linked oxygen of the second sugar and C6 of the third sugar. Meanwhile, the side chain of D231 is positioned to make hydrophobic contacts with C4 of the sixth sugar moiety. Although our ESI-MS data suggested that mutation of R140 does not affect binding, a hydrogen bond is predicted between the guanidinium group of this residue and the C6 oxygen of the hydroxymethyl side chain of the third sugar as well as the carbonyl oxygen of the acetamido group of the fourth sugar. Collectively these *in silico* findings support our experimental observations and suggest that the CBM binding cleft, which spans roughly six subsites, provides an extended polymer binding groove, thereby allowing PelA_DA_ to bind longer oligosaccharides (Fig. S9*C*).

### PelA_DA_ is predicted to form a cytoplasmic membrane complex with PelG

Exopolysaccharide biosynthesis typically involves a complex of two or more proteins that work in unison to synthesize and export the polymer to a desired cellular destination. In *P. aeruginosa*, PelD, PelE, PelF and PelG have been shown to directly interact with each other (15). Given that *B. cereus* ATCC 10987 homologs of PelD, PelE, PelF and PelG share 19 to 33% sequence similarity to *P. aeruginosa*, we hypothesize that a similar complex of PelDEFG would form in the cytoplasmic membrane of *B. cereus* ATCC 10987 to facilitate Pel polymerization and export into the extracellular space. Since the presence and catalytic activity of *B. cereus* ATCC 10987 PelA_DA_ is required for Pel biosynthesis, this also suggests a link between intracellular polymer synthesis and extracellular polymer modification *via* an interaction with the PelDEFG complex. Within the PelDEFG complex, the export apparatus PelG will determine how the nascent Pel polymer is presented for deacetylation by PelA_DA_. To gain insight into whether PelA_DA_ can associate and form a complex with the cytoplasmic membrane Pel machinery in *B. cereus* ATCC 10987, we generated *in silico* heterocomplexes of monomeric PelA_DA_ and PelG using AF2 and AF3.

**Figure 6:**
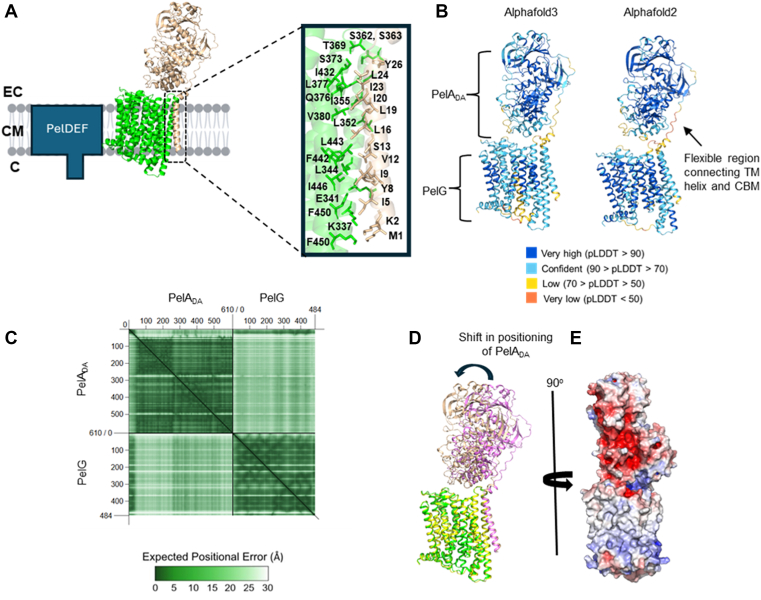
**Alphafold2 and Alphafold3 predictions indicate the formation of a PelA_DA_-PelG inner membrane complex.***A*, cartoon representation of the PelA_DA_ (*wheat*) and PelG (*green*) complex as predicted by AF3. The predicted PelDEF cytoplasmic membrane complex is shown in *blue*. The M1 helix of PelA_DA_ interacts with α9, 10 and 12 of PelG. Residues hypothesized to be involved in PelA_DA_-PelG interactions are labelled and depicted as *sticks*. *B*, cartoon representation of the PelA_DA_-PelG AF2 and AF3 complexes colored based on predicted per-residue confidence score (pLDDT). *C*, Residue–residue predicted alignment error (PAE) plot for the AF3 PelA_DA_-PelG prediction. The values range from 0 to 30 Å. *D*, Superimposition of the Alphafold2 (*yellow*) and Alphafold3 (*green*) PelG models with PelA_DA_. The Alphafold2 and Alphafold3 PelA_DA_ predictions are colored *pink* and wheat, respectively. *E*, surface electrostatic representation of the AF3 PelG and PelA_DA_ model. Electrostatics were calculated by APBS in PyMol (v. 2.4) and visualized in blue to red (+5 kT/e to −5 kT/e). C, cytoplasm; CM, cytoplasmic membrane; EC, extracellular space.

In the AF2 and AF3 models, the N-terminal residues of PelA_DA_ (residues 1–26) are predicted to adopt a membrane-embedded hydrophobic helix (M1-helix) which aligns with results from the DeepTMHMM topology prediction server (45). PelG belongs to the multidrug/oligosaccharidyl-lipid/polysaccharide exporter superfamily and adopts a 12-helix bundle (Fig. 6*A*) (46). In the AF3 model, the M1-helix of PelA_DA_ predominantly interacts with helices α9,10 and 12 of PelG. 13 PelA_DA_ and 18 PelG residues are located within 4 Å of each other, facilitating the formation of a PelA_DA_-PelG complex (Fig. 6, *A* and *C*). Residues 28 to 46 of PelA_DA_, which were not modeled in our crystal structure, are predicted with low confidence in both models (Fig. 6*B*). This region is predicted to adopt a continuous coil (Fig. 6*B*), which allows PelA_DA_ to adopt an orientation with the CE domain proximal to the membrane and the CBM and β-rich domains oriented towards the peptidoglycan layer (Fig. 6, *A–C*). The positioning of the CE domain proximal to the membrane may also create contacts between PelA_DA_ and PelG along the plane of the membrane. Superimposition of PelG between the two models reveals a shift in the orientation of PelA_DA_ mediated by the residues 28 to 46 (Fig. 6, *D* and *E*). This variability suggests that the PelA_DA_-PelG complex may adopt multiple conformations influenced by the positioning of the N-terminal residues of PelA_DA_. The orientation of PelA_DA_ in both models also support a direct connection between PelG and PelA (Fig. 6). As modelled, the CE domain is positioned to receive the fully acetylated Pel polymer as it exits through the PelG pore. Our model also suggests that the CBM binding would occur once the polymer is deacetylated by the CE domain. The orientation of the CE and CBM domains relative to PelG is supported by our ESI-MS results which suggest that the CBM can bind both fully acetylated and partially deacetylated GalNAc oligomers. Overall, our model suggests that in *B. cereus* ATCC 10987, PelA_DA_ interacts with PelG, and that as the polymer exits through the pore in PelG, it is immediately partially deacetylated prior to its release into the extracellular space.

## Discussion

In this study, we demonstrate that the structure of a novel polysaccharide deacetylase, PelA_DA_, has a three-domain arrangement reminiscent of the GAG deacetylase Agd3 (20). Using a combination of enzyme assays and MALDI-TOF MS analyses, we establish that PelA_DA_ deacetylates α-1,4-GalNAc oligosaccharides. Despite its structural and mechanistic similarities to CE4, CE18 and CE21 enzymes, its sequence divergence is high enough that the CE domain of PelA_DA_ does not fall into any existing CE family. Similarly, the CBM does not belong to any known class of CBM. Combined, our structural and functional characterization of PelA_DA_ resulted in the enzyme being classified as the founding member of the CE26 and CBM114 families. We found that the deacetylase activity of PelA_DA_ is essential for the biosynthesis of Pel and Pel-dependent biofilm formation and suggest a model for how polymer export and extracellular deacetylation are linked through a direct interaction between PelA_DA_ and PelG.

Members of CE families 4, 9, 14, 18, and 21 require the presence of a divalent cation for activity (31, 33). Unlike previously characterized CE enzymes, a strict metal dependence could not be demonstrated as the esterase activity of PelA_DA_ was not inhibited with the addition of the chelators EDTA and DPA (Fig. 2*C*). The inability of EDTA to abolish the esterase activity of CE4, CE18 and CE21 enzymes has been reported previously and is attributed to low diffusion of metal ions from the active site and the large size of EDTA (20, 37). In our structural analysis, we observed notable differences in the coordination of Zn^2+^ ions in the active sites of chains A and B. These variations could arise from the conformational flexibility of the MT2 catalytic acid H488, differential effects of crystal packing or the dynamic nature of water molecules near the Zn^2+^ ions. While we have modeled in Zn^2+^ in the active site, it is important to note that promiscuity in metal binding is often observed among CEs. CE4 enzyme PgaB, for instance, demonstrates an increase in activity upon addition of Ni^2+^ and Co^2+^ while CE9 ureases can bind one or more metal ions, including Fe^2+^ and Cu^2+^ (33, 37, 47). Furthermore, CE4 enzymes can have metal-induced increases in activity against certain substrates and not others. For example, xylan esterase and chitin deacetylases are potentiated by Co^2+^ only with soluble chitinous substrates (48, 49). Our studies demonstrate that the addition of Mn^2+^ and Cu^2+^ increases the activity of PelA_DA_. Mn^2+^, in particular, is critical for bacterial pathogenesis and colonization of environments. In *Bacillus subtilis*, Mn^2+^ exposure is linked to the activation of a gene expression cascade responsible for exopolysaccharide biosynthesis and promotes sporulation efficiency, swarming, resistance to disinfectant, and biofilm formation (50, 51, 52). The regulatory pathways governing Mn^2+^-dependent biofilm formation of *B. cereus* ATCC 10987 have yet to be explored, but our results suggest that the extent of Pel deacetylation could depend on extracellular concentrations of divalent cations.

In addition to the catalytic core, comprised of residues in MT1, two and 5, the side chains of key residues in MT3 and MT4 determine the catalytic efficiency of CE enzymes (37, 53). In PelA_DA,_ differences in two key residues relative to canonical CE4, CE18 and CE21 enzymes were identified. First, in CE4 and CE18 enzymes a critical acidic residue in MT4 activates the catalytic acid. Enzymes that lack this key residue, such as PgaB and Agd3, typically use a water molecule as a substitute for the aspartic acid, which lowers the catalytic efficiency (20, 37). PelA_DA_ lacks this conserved acidic residue as structural comparisons with PgdA indicate that the analogous region is occupied by a short β/α loop comprised of hydrophobic residues. Instead, we identified an equivalent residue, D491, located at the end of MT5/CM4 that could activate H488 thereby lowering its pKa (Fig. 2*B*). Secondly, CE4/CE18 enzymes also possess a conserved MT3 arginine that activates the catalytic base. In PelA_DA_ this conserved arginine occurs later in the primary structure and was identified to be R458. In IcaB, the equivalent arginine is locked in a bi-dentate salt bridge with an MT1 aspartic acid and as a consequence, the MT5 histidine functions as a bifunctional acid-base catalyst in the enzymatic mechanism (29). R458 in PelA_DA_ is oriented to form a similar bi-dentate salt bridge with the catalytic base D262. However, the requirement of R458 and D262 for catalytic activity as depicted by Pel dots blots (Fig. 4, *B* and *E*) suggests that the catalytic mechanism is not the same as IcaB. Thus, in our proposed enzymatic scheme, R458 plays a role similar to canonical CE4 enzymes wherein it activates the catalytic base D262 that extracts a proton from a neighboring Zn^2+^ coordinated water molecule. The nucleophilic hydroxide ion would then attack the carbonyl group on the bound polysaccharide's acetate group at the 0 subsite, creating an oxyanion intermediate. H488 would then act as a proton donor, releasing the acetate group, and completing a single deacetylation event (Fig. 2*E*).

Accessory domains are typically part of exopolysaccharide modification enzymes and assist in improving substrate engagement and bioavailability (54). CBMs are often associated with glycoside hydrolases and are important in accelerating substrate degradation (54). Among CE4 enzymes, chitin deacetylases (CDAs) have one or more CBMs fused to the catalytic domain, and these domains play a vital role in increasing substrate accessibility (40, 49). The CDA from *Podospora anserina* also has a canonical CE4 domain flanked by two CBM18 domains, which help the enzyme act on insoluble substrates (55). ESI-MS data with hexamers of GalNAc indicate that the N-terminal domain of PelA_DA_ is a CBM that can bind both fully and partially deacetylated oligosaccharides. This suggests that Pel binding to the CBM may occur after deacetylation (Fig. 5*C*).

The observed deacetylase activity on GalNAc oligomers *in vitro* is consistent with the predicted biological role of PelA_DA._ While the precise composition and chemical structure of *B. cereus* ATCC 10987 Pel has not yet been determined, insights can be obtained from *P. aeruginosa*, where the deacetylase activity of PelA results in a polymer comprised predominantly of dimeric repeats of GalNAc and GalN (10). In *P. aeruginosa*, Pel is not detected in a PelA deletion strain or a mutant that lacks deacetylase activity (19, 36). Our data is consistent with this phenotype as we demonstrate using our monoclonal antibody raised against GalNAc_3_ that polymer is not detected in either the cell-associated or cell-free fractions of the *ΔpelA*_*DA*_ strain or when this deletion mutant is complemented with catalytically inactive or substrate binding mutants of PelA_DA_. We hypothesize that polymer deacetylation by PelA_DA_ is critical for regulating biosynthesis by the cytoplasmic membrane machinery and that the inability to deacetylate the polymer could act as a cue to halt biosynthesis.

A key similarity between Pel systems of *P. aeruginosa* and *B. cereus* ATCC 10987 is the homology between the inner membrane and cytoplasmic membrane biosynthetic machinery, PelDEFG. While the details of inner membrane Pel protein complex assembly in *P. aeruginosa* have been studied (15), how these proteins in Gram-negative species link to Pel deacetylation by PelA and subsequent export by PelB and PelC is unclear. In *B. cereus* ATCC 10987, the transmembrane domain of PelA_DA_ enables the protein to be directly associated with Pel biosynthetic machinery. Using AF2/AF3 modelling we determined a structural model of the PelA_DA_-PelG complex (Fig. 6*B*) which provides a framework for understanding the coupling of Pel export and modification. The model suggests that PelG, in addition to acting as part of the Pel export machinery, interacts with PelA_DA_. The PelG-PelA_DA_ interaction is primarily driven by transmembrane contacts between the M1 helix of PelA_DA_ and C-terminal helices (α9,10 and 12) of PelG. Given the inherent flexibility of residues 28 to 62, it is hard to predict with any certainty the orientation of PelA_DA_ relative to PelG, but the model does suggest that Pel export into the extracellular space and modulating the spatial orientations of the CBM and CE domains to facilitate deacetylation would be feasible. We hypothesize that the activity of PelA_DA_, or the lack thereof, can dictate polymer biosynthesis by the glycosyltransferase, PelF, *via* a feedback mechanism that relays through PelG. This mode of regulation would be beneficial to the bacterium to reduce the metabolic burden and prevent the accumulation of acetylated Pel. Future studies deciphering the mechanisms that regulate deacetylation and export will enhance our understanding of Pel biosynthesis in *B. cereus* ATCC 10987 and other Gram-positive Pel-producing organisms.

## Experimental procedures

### Tools for computational analysis

Amino acid sequence of PelA_DA_ (BCE_5585) was obtained from UniProt and analyzed using the PSIPRED Workbench (56). The DALI server was used to search for structurally similar proteins (57). Amino acid conservation among residues was calculated by Consurf (58). Electrostatic potential maps were calculated with APBS and visualized in PyMOL (The PyMOL Molecular Graphics System, Version 1.2, Schrödinger, LLC) (59). Structural superimpositions were performed using the ‘super’ function in PyMOL. This method performs a sequence-independent alignment followed by five cycles of iterative refinement and reports an RMSD value between the two alignments. Multiple sequence alignments were performed using the Clustal Omega server (60). Structural predictions of PelA_DA_-PelG were generated using AF2 (ColabFoldv.1.5.2) and the AF3 web server (https://alphafoldserver.com) with default parameters (61, 62, 63). Five AF2 and AF3 models each were generated and the highest confidence model, as assessed by pLDDT scores and PAE plots, was selected for analysis. PelA_DA_-polymer predictions were generated using AF3 (available at https://github.com/google-deepmind/alphafold3), which was run on a local server (64). The AF3 model parameters file was obtained from the DeepMind team. The PelA_DA_ protein construct used included residues 63 to 254. AF3 modelling was performed following the protocol outlined by Huang *et al.* (2025), using the Chemical Component Dictionary (CCD)-based ligand specification and *bondedAtomPairs* (*BAP*) syntax to define glycosidic linkages in the GalNAc hexamer (65).

### Bacterial strains and growth conditions

A detailed list of all bacterial strains and plasmids used in this study can be found in Table S2. Unless otherwise stated, *Bacillus* strains were grown in lysogeny broth (LB) containing 10 g tryptone, 5 g NaCl, and 10 g yeast extract per liter of deionized ultrapure water.

### Generation of recombinant protein constructs

To generate the expression construct for PelA_DA,_ the open reading frame of PelA_DA_ (excluding the predicted N-terminal transmembrane helix (residues 1–26)) was amplified from *B. cereus* ATCC 10987 genomic DNA with the BestTaq polymerase (Applied Biological Materials Inc.). Primers (Table S2) were designed with restriction sites for ligation-dependent cloning into the pET24a vector, which encodes a C-terminal hexahistidine tag. The resulting vector pET24a-PelA_DA_^27-610^ was transformed into *E. coli* Top10 and selected on LB agar containing 50 μg/ml kanamycin. Point mutants of PelA_DA_^27-610^ were generated using the QuickChange Lightning site-directed mutagenesis kit (Agilent) and the Phusion polymerase (New England Biolabs) with pET24a-PelA_DA_^27-610^ as the template. Round the horn mutagenesis generated PelA_DA_^27-253^. Plasmids were verified by Sanger sequencing using the T7 primer at The Center for Applied Genomics (TCAG).

### Expression and purification of PelA_DA_^27-610^ and PelA_DA_^27-253^

All constructs were transformed into chemically competent *E. coli* BL21-CodonPlus cells. A single colony was used to inoculate 50 ml of LB and grown overnight with shaking (200 rpm). The overnight cultures were added to 2 L of Terrific broth (TB) containing 47.6 g of terrific broth and 0.4% (w/v) glycerol per litre of deionized ultrapure water and cells were grown at 37 °C until the OD_600_ of the culture was 0.6 to 0.8. Isopropyl-β-D-1-thiogalactopyranoside (IPTG) was added to a final concentration of 1 mM to induce gene expression and the cells were incubated at 18 °C for 24 h. Cells were harvested by centrifugation at 5500*g* for 20 min at 4 °C and pellets were frozen at −20 °C until used for purification.

To purify proteins for *in vitro* assays, cell pellets were thawed at 25 °C and resuspended in 40 ml Lysis Buffer A [50 mM HEPES pH 8, 300 mM NaCl and 10% (v/v) glycerol, 20 mM imidazole pH 8] with one protease inhibitor tablet (Thermo Fisher Scientific). Cells were lysed by sonication (Misonix 3000) on an ice bath at 65% output for 1 min and 30 s. The cell lysate was clarified by centrifugation at 25,000*g* for 40 min at 4 °C. The supernatant was loaded onto a gravity column containing 1 ml of Ni-NTA resin pre-equilibrated with Lysis Buffer A and incubated for 30 min at 4 °C with gentle agitation. The resin was washed with 40 ml of lysis buffer and twice with 40 ml of wash buffer [50 mM HEPES pH 8, 300 mM NaCl and 10% (v/v) glycerol, 40 mM imidazole pH 8] to remove unbound proteins. Protein was eluted using 10 ml of Elution Buffer A [50 mM HEPES pH 8, 300 mM NaCl and 10% (v/v) glycerol, 60 mM imidazole pH 8] and 10 ml of Elution Buffer B [50 mM HEPES pH 8, 300 mM NaCl and 10% (v/v) glycerol, 100 mM imidazole pH 8]. The two elutions were combined and concentrated to 2 ml using a 30 kDa cut-off centrifugation filter unit (Amicon) and further purified using size exclusion chromatography (SEC) with a HiLoad 16/60 Superdex 200 gel filtration column (GE Healthcare) into Buffer C [50 mM HEPES pH 8, 300 mM NaCl and 5% (v/v) glycerol]. For the purification of PelA_DA_^27-610^ for crystallization, the cell pellets were lysed in Lysis buffer B [50 mM HEPES, pH 7, 50 mM NaCl and 10% (v/v) glycerol, 20 mM imidazole, pH 8]. Following nickel purification, purified protein was dialyzed into Buffer C and SEC purification was carried out as stated above. The following yields of purified protein were obtained: wild-type *PelA*_*DA*_^*2-7-610*^, 1.51 mg and the *PelA*_*DA*_^*2-7-610*^ mutants: H350A, 0.95 mg; H354A, 1.44 mg; P405A, 1.54 mg; H488A, 1.86 mg; wild type *PelA*_*DA*_^*27-253*^, 2.1 mg; and the *PelA*_*DA*_^*27-253*^ D231A, 2.26 mg; E115A, 2.6 mg; R140A, 2.02 mg; W147A, 0.53 mg; and E218A, 1.87 mg. The protein concentration was calculated using a NanoDrop spectrophotometer (Thermo Scientific).

### Crystallization, data collection, refinement, and model building

Purified PelA_DA_^27-610^ was subjected to crystallization screening using the sitting-drop vapor diffusion method using the Oryx8 crystallization robot (Douglas Instruments) at 4 mg/ml and 2 mg/ml. Crystals formed in 0.2 M ammonium fluoride, 20% (v/v) PEG3350 (Microlytic MCSG1 condition 85). Optimization screens were set up by varying the concentration of ammonium fluoride and PEG3350. Small crystals were obtained in 0.15 M Ammonium fluoride, 27.5% (v/v) PEG3350. Crystals were harvested, soaked in mother liquor supplemented with 200 mM ZnSO_4_, and transferred to a cryoprotectant solution before vitrification in liquid nitrogen. Diffraction data were collected at beamline 17ID, NSLS-II. 360° of data were measured at 1° ϕ oscillation to 2.51 Å. The dataset was collected at the Zn^2+^ absorption edge to facilitate single-wavelength anomalous diffraction (SAD) phasing. The data were indexed, merged, and scaled using XDS (66). Subsequent analysis revealed insufficient anomalous signal for phasing and the structure was therefore determined by molecular replacement in Phaser using an AF2 structure prediction of PelA_DA_, downloaded from UniProt, as the search model (61, 67, 68). The initial model was refined using rigid body and restrained refinement in REFMAC5 followed PHENIX.REFINE (69, 70). The structure was validated using omit maps created using PHENIX.REFINE. 10 TLS groups were added using the TSLMD server in Phenix. Iterative refinement in combination with manual adjustments and addition of water molecules in COOT (71, 72, 73, 74, 75) resulted in a model with an *R*_factor_ and *R*_free_ of 19.41% and 25.63%, respectively (Table 1).

### AMMU esterase assay

This assay was performed as described previously (36). Briefly, in a 96-well polystyrene V-bottom mixing plate 30 μM of PelA_DA_^27-610^ was incubated with equimolar amounts of indicated metal chloride solutions for 30 min at 25 °C. AMMU prepared in a 50:50 mixture of DMSO and Buffer B was added to a final concentration of 225 μM and incubated at 25 °C for 1.5 h. Reactions were transferred to a 384-well, black flat-bottom assay plate (Costar; 3573) and centrifuged at 2000*g* for 30 s. Fluorescence measurements were recorded using a Synergy Neo2 (BioTek) plate reader at an λ_em_ of 330 nm and λ_ext_ of 450 nm. Data were normalized to the protein-only control.

### Fluorescamine assay

30 μM of PelA_DA_^27-610^ wild-type and mutants were prepared in a buffer containing 50 mM HEPES pH 8, 300 mM NaCl and 5% (v/v) glycerol. 30 μM of Mn^2+^ was added to each reaction followed by the addition of α-1,4-(GalNAc)_9_ to a final concentration of 5 mM in 25 μl reactions. Reactions were incubated for 24 h at 20 °C. 10 μl samples were added to 20 μl of 0.5 M borate buffer, pH 9.0, and 10 μl of a freshly prepared 20 mg/ml fluorescamine solution in dimethylformamide. Reactions were quenched by adding 80 μl deionized water, and fluorescence measurements were performed using the Synergy Neo2 (BioTek) plate reader at an λ_em_ of 360 nm and λ_em_ of 460 nm. Glucosamine solutions were used as standards to calculate amine concentrations.

### Crystal violet microtiter plate assay

Crystal violet adhesion assays were performed as described previously (14). Briefly, overnight cultures of *B. cereus* ATCC 10987 strains were grown in LB supplemented with 10 μg/ml chloramphenicol. Cultures were subsequently normalized to an OD_600_ of 0.5, plated onto Corning CellBind 96-well microtiter plates in the presence of 10 μg/ml chloramphenicol and 1% (w/v) xylose and incubated at 30 °C in a humidified incubator. After 24 h, nonadherent cells were removed by gently washing the plates twice with 200 μl of deionized water followed by the addition of 125 μl of 0.1% (w/v) crystal violet stain for 30 min at room temperature with shaking. The plates were then gently rinsed three times with 200 μl deionized water and left to air dry for 6 to 8 h. The adhered crystal violet was solubilized with 100 μl of 30% (v/v) acetic acid for 10 min and absorbance was measured at 550 nm.

### Complementation of *B. cereus* ATCC 10987 gene deletions

For complementation, point mutants of PelA_DA_ were generated using the QuickChange Lightning site-directed mutagenesis kit (Agilent) and the Phusion polymerase (New England Biolabs) with pAD123-P_xyl_-*pelA*_*DA*_^*27-610*^ as the template. These plasmids were transformed into chemically competent *E. coli* EC135 containing plasmid pM.Bce. Transformed EC135 strains were plated on LB agar containing 100 μg/ml spectinomycin and 100 μg/ml carbenicillin and incubated at 30 °C for 24 h. 5 ml of LB was inoculated with single colony transformants and the desired pAD123-P_xyl_ plasmids were methylated overnight at 30 °C by the addition of 0.2% (w/v) L-arabinose. Methylated plasmids were isolated and transformed into electrocompetent *B. cereus* ATCC 10987 *ΔpelA*_*DA*._ To generate electrocompetent *B. cereus* ATCC 10987 *ΔpelA*_*DA*,_ 5 ml of overnight culture was grown to saturation. 0.2 ml of overnight culture was used to inoculate 50 ml of LB and grown to an OD_600_ of 0.4. Cells were harvested by centrifugation at 4300*g* for 10 min at 4 °C and washed five times in electroporation buffer containing 1 mM Tris pH 8.0, 10% (w/v) sucrose, 15% (v/v) glycerol. Cells were finally resuspended in 0.8 ml of electroporation buffer and used immediately. 1 μg of methylated plasmid was added to 100 μl of electrocompetent *B. cereus* ATCC 10987 *ΔpelA*_*DA*_ and incubated on ice for 10 min. The mixture was transferred to a chilled 2 mm gap electroporation cuvette (Bio-Rad) and incubated on ice for 10 min. Cells were then pulsed once using a BioRad Gene Pulser electroporator with capacitance extender at 2.5 kV, 200 Ω, and 25 μF, followed by immediate addition of 1 ml of NCMLB medium (100 mM K_2_HPO_4_, 200 mM NaCl, 30 mM glucose, 10 mg/ml tryptone, 5 mg/ml yeast extract, 1 mM trisodium citrate, 0.2 mM MgSO_4_, 380 mM mannitol, 500 mM sorbitol, pH 7.2). Cells were grown shaking at 37 °C for 4 to 5 h following which 200 μl was plated onto LB-Agar plates supplemented with 10 μg/ml chloramphenicol.

### PelA_DA_ antibody generation and purification

PelA_DA_^27-610^ was purified as described above. Anti-serum from rabbits were generated using a standard 70-days protocol (Cedarlane Laboratories). To purify the α-PelA_DA_ antibody, 50 μg of purified PelA_DA_^27-610^ was loaded onto five lanes of a 12% SDS-PAGE gel and transferred to a polyvinylidene difluoride (PVDF) membrane. The membrane was then stained with Ponceau S and upon development, corresponding protein bands were excised and washed with deionized water to remove excess stain. Membrane pieces were blocked in phosphate buffered saline (PBS) pH 7 with 0.1% (w/v) Tween-20 and 5% (w/v) milk powder for 1 h. Membrane pieces were incubated with anti-sera for 15 h at 4 °C and subsequently for 2 h at 25 °C. Following three washes in PBS + 0.1% (w/v) Tween-20 for 15 min each, α-PelA_DA_ was eluted by the addition of 700 μl 0.2 M glycine pH 2.2 for 15 min and neutralized with 300 ml 1 M K_2_HPO_4_. The mixture was dialyzed for 24 h in PBS at 4 °C, combined in a 1:1 ratio with 100% (v/v) glycerol, and stored at −20 °C until use.

### Dot blots

Cell-associated and cell-free Pel fractions were isolated as described previously (14). Once isolated, 5 μl of cell-associated and secreted Pel, were pipetted onto a nitrocellulose membrane and left to air dry for 1 h. The membrane was blocked with 5% (w/v) skim milk in Tris-buffered saline with Tween-20 (TBS-T) for 1 h at room temperature with shaking. Blots were probed with mouse α-(GalNAc)_3_ monoclonal antibody at a 1:2000 dilution in 1% (w/v) skim milk prepared in TBS-T overnight at 4 °C with shaking (43). Blots were washed three times for 15 min each with TBS-T and probed with goat α-mouse HRP-conjugated secondary antibody (Bio-Rad) at a 1:2000 dilution for 1 h at room temperature with shaking. Blots were then washed three times and developed using the SuperSignal West Pico (Thermo Fisher Scientific) kit.

### MALDI-TOF MS analysis of deacetylation

Monitoring of α-1,4-(GalNAc) oligosaccharides deacetylation was performed as previously reported (20). Briefly, α-1,4-(GalNAc) oligosaccharides were extracted and purified from galactosaminogalactan produced within *A. fumigatus* biofilms. The deacetylation reaction was performed using 30 μM of PelA_DA_^27-610^ wild-type or mutants in a buffer containing 50 mM HEPES pH 8, 300 mM NaCl, 5% (v/v) glycerol and 30 μM of MnCl_2_ for 24 h at 37 °C. Oligosaccharides were then purified with a HyperSep HyperCarb column using 50% (v/v) acetonitrile (ACN) as the elution buffer. Eluates were dried, reconstituted in 10 μl 0.2% TFA and spotted on the MALDI-TOF plate in DHB matrix. Data acquisition was performed on a UltrafleXtreme Bruker MALDI-TOF mass spectrometer using a Reflectron positive mode.

### ESI-MS binding assay

Purified PelA_DA_^27-253^ was buffer exchanged into 200 mM aqueous ammonium acetate (pH 8.0) using a 3 kDa cut-off Amicon 0.5 ml microconcentrator (EMD, Millipore). Synthesized oligosaccharide ligands were purified using GlycoClean S cartridges (Agilent Technologies, CA). Stock solutions of oligosaccharides were prepared by dissolving known amount of solid compound in milliQ water and stored at −20 °C until used (76). Nanoelectrospray (nanoESI) mass spectrometry measurements were performed using Q Exactive Orbitrap (Orbitrap) mass spectrometer (Thermo Fisher Scientific, Bremen, Germany), equipped with a nanoflow ESI source. NanoESI was performed by applying a voltage of ∼0.8 kV to a platinum wire inserted into the nanoESI tip, which was produced from a borosilicate glass capillary (1.0 mm o.d., 0.78 mm i.d.) pulled to ∼2 μm o.d. using a *p* − 1000 micropipette puller (Sutter Instruments). The inlet capillary of the MS was heated to 120 °C, S-lens RF level was set at 100, automatic gain control target set at 1 × 10^6^ with a maximum injection time of 50 to 200 ms. All MS data were acquired and processed using Thermo Xcalibur 4.1 software.

Dissociation constants (*K*_d_) for interactions of PelA_DA_^27-253^ with oligosaccharide ligands were obtained using the direct ESI-MS assay (77, 78). The binding measurements were carried out at room temperature, the reaction mixtures were prepared by mixing aliquots of the stock solutions of protein and ligands in aqueous 200 mM ammonium acetate. ESI-MS measurements were performed after 1 h incubation. All mass spectra were corrected, when needed, for the occurrence of nonspecific carbohydrate-protein binding during the ESI process using the reference protein streptavidin (P_ref_) (79). *K*_d_ values were determined from the abundance *(Ab)* ratio *(R)* of the ligand-bound *Ab*(PL) to free protein *Ab*(P) ions, after correction for nonspecific ligand binding, and the initial concentrations of protein ([P]_0_) and oligosaccharide ligand ([L]_0_), Equations (1) and (2):

(1) $$K_{d}=\frac{\left[P\right]\left[L\right]}{\left[\text{PL}\right]}=\frac{{\left[L\right]}_{0}}{R}-\frac{{\left[P\right]}_{0}}{R+1}$$

where R is taken to be equal to the corresponding concentration ratio ([PL]/[P]) in solution, Equation (2):

(2) $$R=\frac{\sum Ab\left(PL\right)}{\sum Ab\left(P\right)}=\frac{\left[PL\right]}{\left[P\right]}$$

## Data availability

The coordinates and structure factors for PelA_DA_ have been deposited in the Protein Data Bank, Accession number: 9ZAU.

## Supporting information

This article contains supporting information and cites the following references: (14, 23, 80, 81).

## Conflict of interest

The authors declare that they have no conflicts of interest with the contents of this article.
