# Supplementary material for: Bacillus cereus PelADA is a polysaccharide de-N-acetylase required for pel-dependent biofilm formation
Source: J Biol Chem. 2026 May 7;302(6):113122. doi: 10.1016/j.jbc.2026.113122 (PMC13254593; doi:10.1016/j.jbc.2026.113122)
Supplement: Supporting — Information [file mmc1.pdf]

Supplemental information

Bacillus cereus PelA$_{DA}$ is a polysaccharide de-*N*-acetylase required for Pel-dependent biofilm formation.

Adithya S. Subramanian[1,2], Francois Le Mauff[3,4,5], Elena N. Kitova[6], Roland Pfoh[1], Mayura Panjalingam[1], Dung-Yeh Wu[7], Stephanie Gilbert[1], Zachary A. Morrison[8], Christian Jacobsen-Perez[1,2], Erum Razvi[1,2], Mark Nitz[8], Jeroen Codée[7], John S. Klassen[6], Donald C. Sheppard[3,5,9], P. Lynne Howell[1,2]

[1]Program in Molecular Medicine, The Hospital for Sick Children, Toronto, ON, Canada

[2]Department of Biochemistry, University of Toronto, Toronto, ON, Canada

[3]Infectious Disease and Immunity in Global Health program, Research Institute of the McGill University Health Centre, Montreal, Quebec, Canada

[4]GlycoNet Integrated Services, Microbial Glycomic node, Montreal, Quebec, Canada,

[5]McGill Interdisciplinary Initiative in Infection and Immunity, Montreal, Quebec, Canada

[6]Department of Chemistry, University of Alberta, Edmonton, Alberta, Canada

[7]Institute of Chemistry, Leiden University, Leiden, Netherlands

[8]Department of Chemistry, University of Toronto, Toronto, Ontario, Canada

[9]Department of Microbiology and Immunology, Faculty of Medicine, McGill University, Montreal, Quebec, Canada

Table S1: Melting temperatures of $PelA_{DA}$ WT and point mutants as determined by circular dichroism spectroscopy at 222 nm

**$PelA_{DA}$ enzyme**	**T_m (˚C)**
Wild-type	47.75
H350A	44.73
H354A	47.65
P405A	47.93
H488A	47.89

Table S2: Strains, plasmids and primers used in this study

Strain/Plasmid	Description	Source
***E. coli* strains**		
Top10	Cloning strain; F - *mcrA* (*mrr-hsd*RMS-*mcr*BC) ϕ80*lac*Z ΔM15 Δ*lac*X74 *rec*A1 *ara* Δ139 (*ara-leu*)7697 *gal*U *gal*K *rps*L (Str^R) *end*A1 *nup*G	Invitrogen
BL21-CodonPlus	Protein expression strain; F^-, *ompT hsdS* (rB^- mB^-) dcm^+ Tet^R galλ (DE3) endA [*argU proL* Cam^R]	Stratagene
EC135	Strain lacking endogenous restriction modification systems and DNA methyltransferases; TOP10 Δ*dam* Δ*dcm* Δ*hsd* Δ*mcrBC* Δ*mcrA* Δ*mrr*	Zhang et al. (1)
***B. cereus* strains**		
ATCC 10987	Wild-type strain	A.J. Clarke
ATCC 10987 $\Delta pelA_{DA}$	ATCC 10987 with an unmarked, non-polar deletion of $pelA_{DA}$ *(BCE_5585)*	Whitfield et al. (2)
***A. fumigatus* strains**		
Af293	Wild-type pathogenic strain of *A. fumigatus*	Lee et al. (3)
**Recombinant protein expression plasmids**		
pET24a	IPTG-inducible expression vector encoding C-terminal hexahistidine tag, and Kan^R	Novagen
pET24a::$PelA_{DA\ 27\text{-}610}$	pET24a with *B. cereus* ATCC 10987 BCE_5585 corresponding to residues 27-610 (referred to as $PelA_{DA}^{WT}$) fused to a C-terminal hexahistidine tag; Kan^R	This study
pET24a::$PelA_{DA\ 27\text{-}253}$	pET24a with *B. cereus* ATCC 10987 BCE_5585 corresponding to residues 27-253 (referred to as $PelA_{DA}^{CBM}$) fused to a C-terminal hexahistidine tag; Kan^R	This study
pET24a::$PelA_{DA\ D262N}$	pET24a::$PelA_{DA}^{27\text{-}610}$ with a D262N mutation in the $pelA_{DA}$ gene	This study
pET24a::$PelA_{DA\ D263N}$	pET24a::$PelA_{DA}^{27\text{-}610}$ with a D263N mutation in the $pelA_{DA}$ gene	This study
pET24a::$PelA_{DA\ H350A}$	pET24a::$PelA_{DA}^{27\text{-}610}$ with a H350A mutation in the $pelA_{DA}$ gene	This study
pET24a::$PelA_{DA\ H354A}$	pET24a::$PelA_{DA}^{27\text{-}610}$ with a H354A mutation in the $pelA_{DA}$ gene	This study
pET24a::$PelA_{DA\ H488A}$	pET24a::$PelA_{DA}^{27\text{-}610}$ with a H488A mutation in the $pelA_{DA}$ gene	This study
pET24a::$PelA_{DA\ P405A}$	pET24a::$PelA_{DA}^{27\text{-}610}$ with a P405A mutation in the $pelA_{DA}$ gene	This study
pET24a::$PelA_{DA\ D490N}$	pET24a::$PelA_{DA}^{27\text{-}610}$ with a D490N mutation in the $pelA_{DA}$ gene	This study

pET24a::PelA$_{DA}$ D491N	pET24a::PelA$_{DA}^{27-610}$ with a D491N mutation in the *pelA$_{DA}$* gene	This study
pET24a::PelA$_{DA}$ D231A	pET24a::PelA$_{DA}^{27-253}$ with a D231A mutation in the *pelA$_{DA}$* gene	This study
pET24a::PelA$_{DA}$ E115A	pET24a::PelA$_{DA}^{27-253}$ with a E115A mutation in the *pelA$_{DA}$* gene	This study
pET24a::PelA$_{DA}$ W147A	pET24a::PelA$_{DA}^{27-253}$ with a W147A mutation in the *pelA$_{DA}$* gene	This study
pET24a::PelA$_{DA}$ R140A	pET24a::PelA$_{DA}^{27-253}$ with a R140A mutation in the *pelA$_{DA}$* gene	This study
pET24a::PelA$_{DA}$ E218A	pET24a::PelA$_{DA}^{27-253}$ with a E218A mutation in the *pelA$_{DA}$* gene	This study
pM.Bce	Arabinose inducible vector to express *B. cereus* ATCC 10987 DNA methyltransferases; Spc^R	Zhang et al. (1)
**Complementation in *B. cereus***		
pAD123-P$_{xyl}$	pAD123 with the *xylR*-P$_{xylA}$ cassette from pHCMC04 cloned between the SacI and BamHI sites of pAD123; contains a multiple cloning site (EcoRV-KpnI-NheI-NotI-SmaI-BamHI) immediately downstream of P$_{xylA}$	Whitfield et al. (2)
pAD123-P$_{xyl}$::*pelA$_{DA}$*	*B. cereus* ATCC 10987 *pelA$_{DA}$* (*BCE_5585*) fused to a synthetic RBS (5'-TAAGGAGGAAGCAGGT-3') cloned between the KpnI and BamHI sites of pAD123-P$_{xyl}$	This study
pAD123-P$_{xyl}$::*pelA$_{DA}^{D262N}$*	pAD123-P$_{xyl}$::*pelA$_{DA}$* with a D262N mutation in the *pelA$_{DA}$* gene	This study
pAD123-P$_{xyl}$::*pelA$_{DA}^{H350A}$*	pAD123-P$_{xyl}$::*pelA$_{DA}$* with a H350A mutation in the *pelA$_{DA}$* gene	This study
pAD123-P$_{xyl}$::*pelA$_{DA}^{H354A}$*	pAD123-P$_{xyl}$::*pelA$_{DA}$* with a H354A mutation in the *pelA$_{DA}$* gene	This study
pAD123-P$_{xyl}$::*pelA$_{DA}^{H488A}$*	pAD123-P$_{xyl}$::*pelA$_{DA}$* with a H488A mutation in the *pelA$_{DA}$* gene	This study
pAD123-P$_{xyl}$::*pelA$_{DA}^{P405A}$*	pAD123-P$_{xyl}$::*pelA$_{DA}$* with a P405A mutation in the *pelA$_{DA}$* gene	This study
pAD123-P$_{xyl}$::*pelA$_{DA}^{V403A}$*	pAD123-P$_{xyl}$::*pelA$_{DA}$* with a V403A mutation in the *pelA$_{DA}$* gene	This study
pAD123-P$_{xyl}$::*pelA$_{DA}^{N407A}$*	pAD123-P$_{xyl}$::*pelA$_{DA}$* with a N407A mutation in the *pelA$_{DA}$* gene	This study
pAD123-P$_{xyl}$::*pelA$_{DA}^{R458A}$*	pAD123-P$_{xyl}$::*pelA$_{DA}$* with a R458A mutation in the *pelA$_{DA}$* gene	This study
pAD123-P$_{xyl}$::*pelA$_{DA}^{D490N}$*	pAD123-P$_{xyl}$::*pelA$_{DA}$* with a D490N mutation in the *pelA$_{DA}$* gene	This study

Kan, kanamycin.

**Primer Name**	**Sequence (5' → 3')**
**Recombinant protein purification**	
PelA$_{DA}$-27-F	GGGGG**CATATG**CGAACAGAAAATTTCTATCAAAAG
PelA$_{DA}$-610-R	CCC**CTCGAG**TACCCCTTTAATTGGAATTGTAG
PelA$_{DA}$-D262N-F	GATTATGTATATCaATGACTTCCCATCTCC
PelA$_{DA}$-D262N-R	GGAGATGGGAAGTCATtGATATACATAATC
PelA$_{DA}$-D263N-F	GATTATGTATATCGATaACTTCCCATCTCC
PelA$_{DA}$-D263N-R	GGAGATGGGAAGTtATCGATATACATAATC
PelA$_{DA}$-H350A-F	GAAATCGGTATTgcTGGCTATAACCATC
PelA$_{DA}$-H350A-R	GATGGTTATAGCCAgcAATACCGATTTC
PelA$_{DA}$-H354A-F	CATGGCTATAACgcTCAACCTTTATTATTACC
PelA$_{DA}$-H354A-R	GGTAATAATAAAGGTTGAgcGTTATAGCCATG
PelA$_{DA}$-H488A-F	TCACATTTCGTCgcTCCAGATGATATCC
PelA$_{DA}$-H488A-R	GGATATCATCTGGAgcGACGAAATGTGA
PelA$_{DA}$-P405A-F	AACCTATGTACCAgCATCCAATATTATAAATACAAC
PelA$_{DA}$-P405A-R	GTTGTATTTATAATATTGGATGcTGGTACATAGGTT
PelA$_{DA}$-D490N-F	CGTCCATCCAaATGATATCCTTGATG
PelA$_{DA}$-D490N-R	CATCAAGGATATCATtTGGATGGACG
PelA$_{DA}$-D491N-F	CGTCCATCCAGATaATATCCTTGATG
PelA$_{DA}$-D491N-R	CATCAAGGATATtATCTGGATGGACG
PelA$_{DA}$-D231A-F	GCATTGAACGcaAAATTAGGTAGAG
PelA$_{DA}$-D231A-R	CTCTACCTAATTTtgCGTTCAATGC
PelA$_{DA}$-E115A-F	ATTACAGGTGcAATAATGGCG
PelA$_{DA}$-E115A-R	CGCCATTATTgCACCTGTAAT
PelA$_{DA}$-W147A-F	CAGATCCTTCCgcGAACACATTATTTG
PelA$_{DA}$-W147A-R	CAAATAATGTGTTCgcGGAAGGATCTG
PelA$_{DA}$-R140A-F	ATCGCAAACgcACTTGATTCAGATC
PelA$_{DA}$-R140A-R	GATCTGAATCAAGTgcGTTTGCGAT
PelA$_{DA}$-E218A-F	GACTACGGTGcAGGAAAAGTC
PelA$_{DA}$-E218A-R	GACTTTTCCTgCACCGTAGTC
PelA$_{DA}$-V403A-F	GAAAAACTAAAAACCTATGcACCACCATCCAATAT
PelA$_{DA}$-V403A-R	ATATTGGATGGTGGTgCATAGGTTTTTAGTTTTTC
PelA$_{DA}$-N407A-F	CCTATGTACCACCATCCgcTATTATAAATACAACTGG
PelA$_{DA}$-N407A-R	CCAGTTGTATTTATAATAgcGGATGGTGGTACATAGG
PelA$_{DA}$-R458A-F	CACTTCCCAgcTATTACGAGTGG
PelA$_{DA}$-R458A-R	CCACTCGTAATAgcTGGGAAGTG
PelA$_{DA}$-Q253-R	TTGTACTGTAGCGAAAGTCGGG

$PelA_{DA}$-V610-F	CTCGAGCACCACCACC
**Sequencing**	**Sequence (5' → 3')**
T7	TAATACGACTCACTATAGGG
T7ter	GCTAGTTATTGCTCAGCGG

Table S3 M/Z ratios for the MS-MS fragmentation of the ion at m/z 1625.6 presented in Figure S6.

Fragment	WT		P045A		Neutral loss
	m/z	Intensity	m/z	Intensity	
B7*	225,915	432	225,910	528	$HexNAc_6HexN$
B6	386,935	243	386,933	477	$HexNAc_6$
Z6	406,927	327	406,979	477	$HexNAc_6$
B6*	428,954	806	428,937	784	$HexNAc_5HexN$
Z6*	448,985	747	448,938	1042	$HexNAc_5HexN$
B5	590,045	658	589,982	806	$HexNAc_5$
Z5	610,057	717	610,009	748	$HexNAc_5$
B5*	632,045	807	632,015	810	$HexNAc_4HexN$
Z5*	652,039	877	652,017	1148	$HexNAc_4HexN$
B4	793,116	576	793,086	823	$HexNAc_4$
Z4	813,131	1493	813,078	2273	$HexNAc_4$
B4*	835,089	668	835,093	858	$HexNAc_3HexN$
Z4*	855,086	260	855,108	452	$HexNAc_3HexN$
B3	996,200	481	996,126	857	$HexNAc_3$
Z3	1016,234	1459	1016,166	2088	$HexNAc_3$
B3*	1038,16	374	1038,13	302	$HexNAc_2HexN$
Z3*	1058,209	183	1058,194	98	$HexNAc_2HexN$
B2	1199,267	439	1199,245	681	$HexNAc_2$
Z2	1219,336	1003	1219,273	1507	$HexNAc_2$
Z2*	1260,731	220	1261,341	193	HexNAcHexN
B1	1402,424	214	1402,439	376	HexNAc
Z1	1422,475	723	1422,413	1285	HexNAc
-	1625,655	1392	1625,617	2045	-

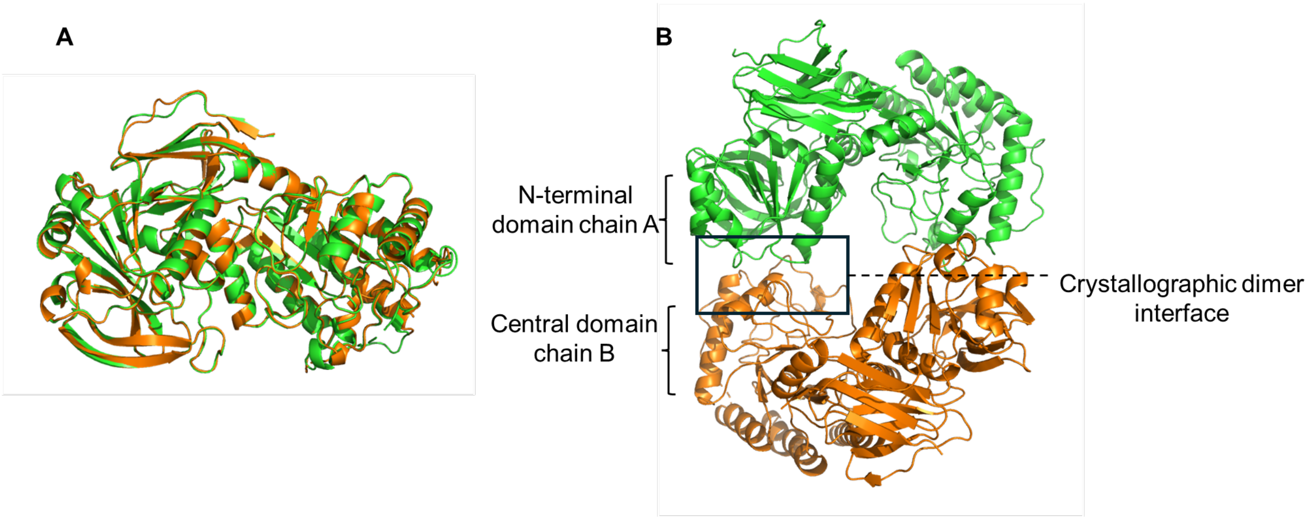

Supplemental Figure 1: Two PelA$_{DA}$ molecules are present in the asymmetric unit. (A) Superimposition of chain A (green) and chain B (orange). RMSD of 0.3 Å over 3481 atoms. (B) Cartoon representation depicting the crystallographic dimer interface of PelA$_{DA}$.

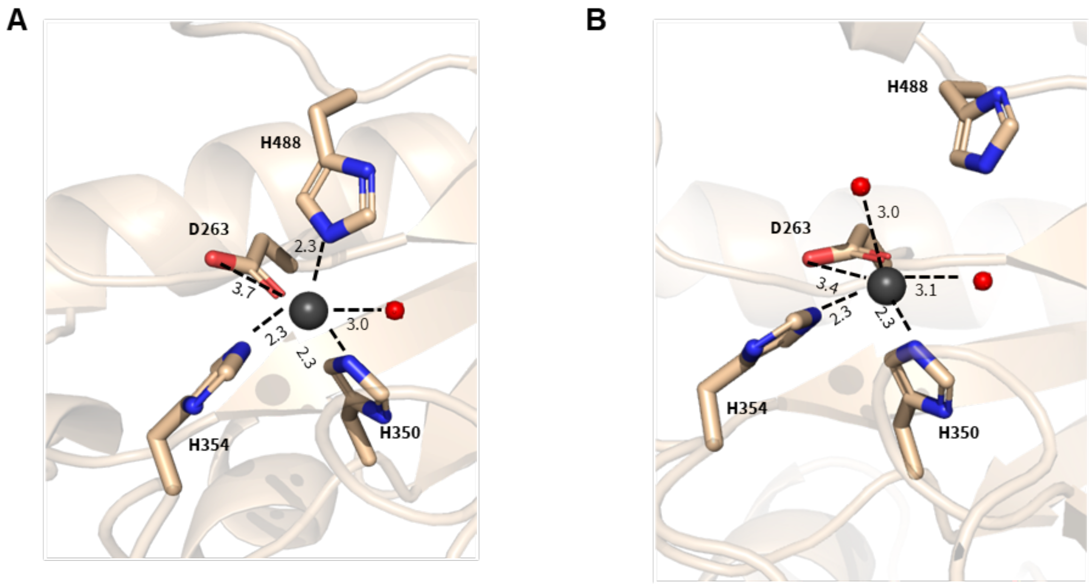

Supplemental Figure 2: Coordination of the active site Zn^{2+} in $PelA_{DA}^{27\text{-}610}$. A distorted trigonal bipyramidal geometry is observed in chain A (A) and B (B). In Chain B H488 is shifted away from the bound Zn^{2+} ion and instead a second water molecule is observed.

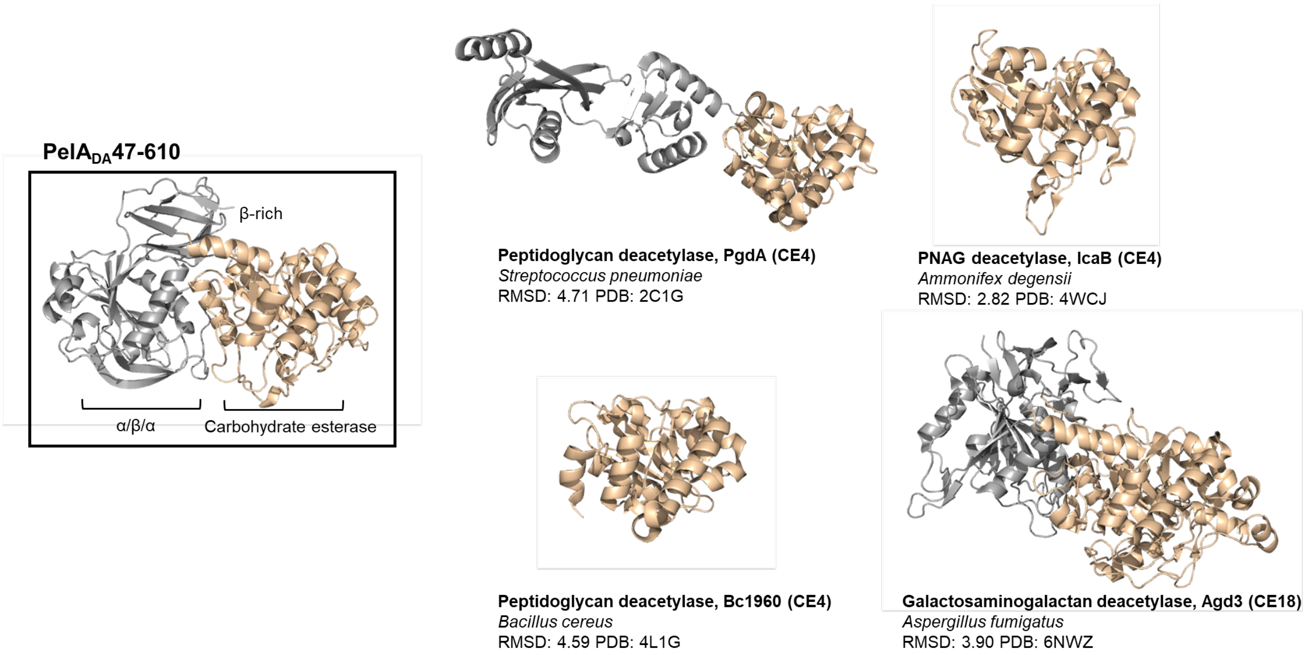

Supplemental Figure 3: DALI server suggests the CE domain of PelA$_{DA}$ is structurally similar to members of the CE4 and CE18 enzymes. Cartoon representation of the top DALI hits with the CE domains of the respective proteins colored in wheat. RMSD values are indicated.

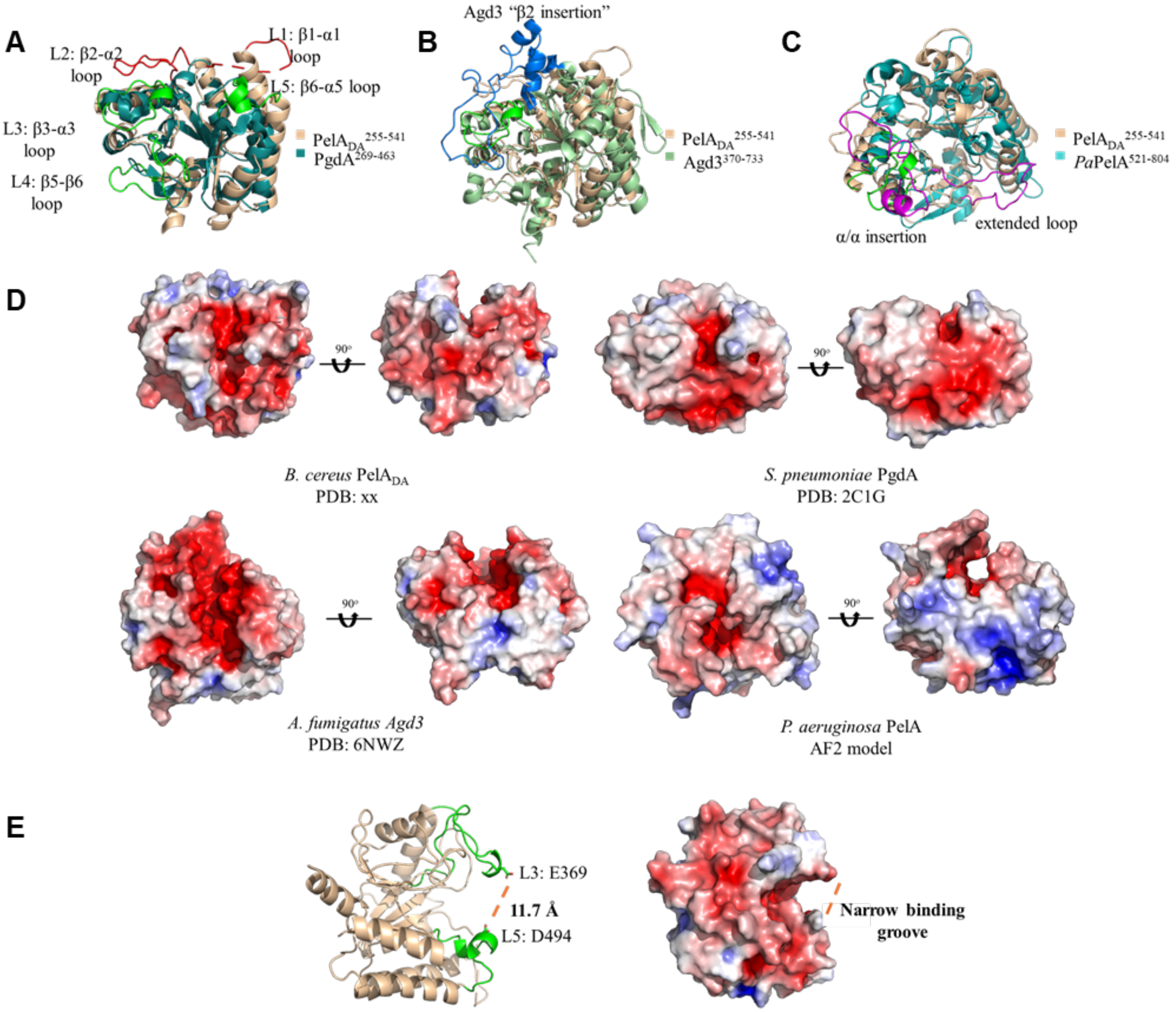

Supplemental figure 4: PelA$_{DA}$ shares structural similarities with CE4, C18 and CE21 family members. Cartoon representation of PelA$_{DA}$ in comparison with (A) *S. pneumoniae* PgdA (dark green), (B*) A. fumigatus* Agd3 (pale green) and (C) *P. aeruginosa* PelA (deep teal). Regions with structural differences between PelA$_{DA}$ and PgdA are highlighted in red and light green. The β2 insertion of Agd3 is colored in blue. The α/α insertion and extended loop of *Pa*PelA is colored in magenta. (D) Electrostatics surface representation depicting the CE domain binding grooves of PelA$_{DA}$, PgdA, Agd3 and *Pa*PelA. (E) Cartoon (left) and electrostatic (right) representation of the narrow CE domain groove of PelA$_{DA}$. For (D) and (E) electrostatics were calculated by APBS in PyMol (v. 2.4) and visualized in blue to red (+5 kT/e to −5 kT/e).

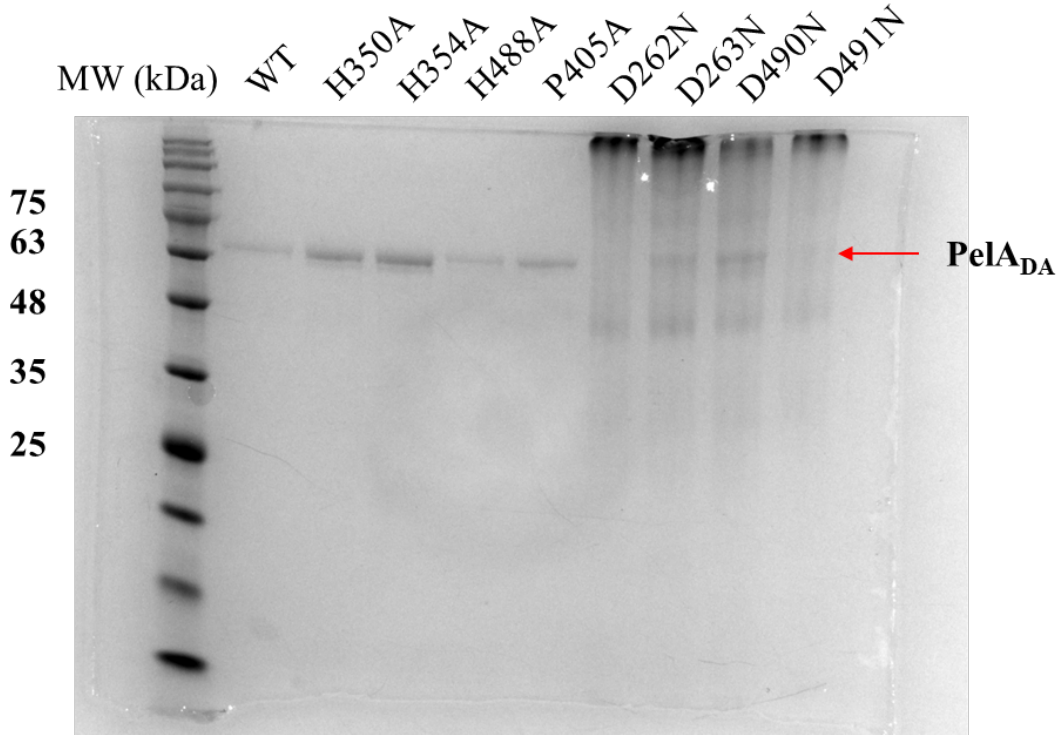

Supplementary Figure 5: Purification and stability of purified PelA$_{DA}$ WT and mutants.
12% SDS-PAGE analysis of SEC200 purified samples of PelA$_{DA}$ WT and mutants. Monomeric PelA$_{DA}$ (indicated with a red arrow) has a molecular weight of 66.7 kDa.

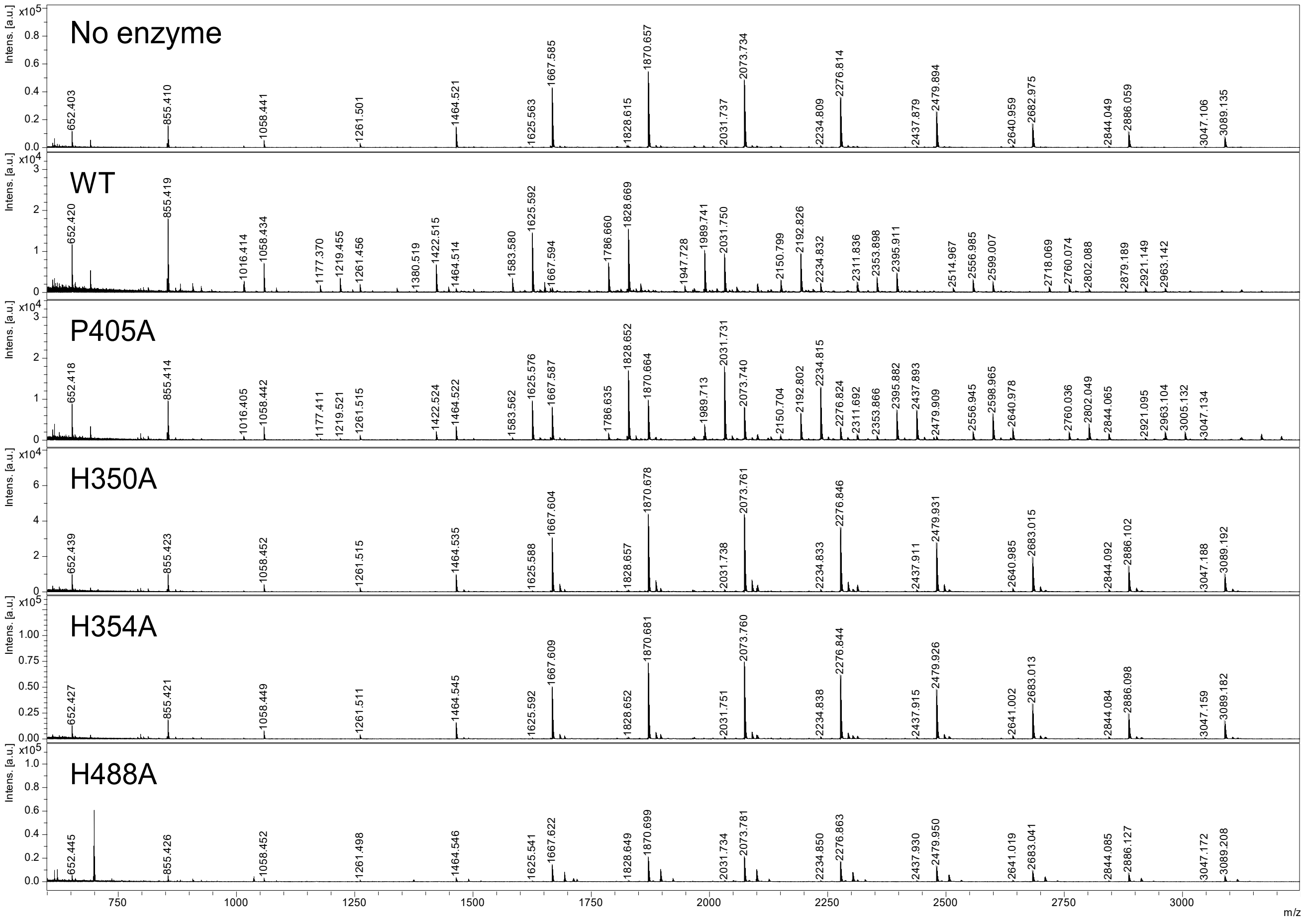

Supplemental figure 6: MALDI-TOF MS supporting data presented in Figure 3. MALDI-TOF MS spectra from m/z 600 to 3250 covering the $HexNAc_3$ to $HexNAc_{15}$ oligomers. All relevant ions in their $[M+Na]^+$ are annotated with their respective m/z. The spectra are representative spectra of the triplicate experiment performed.

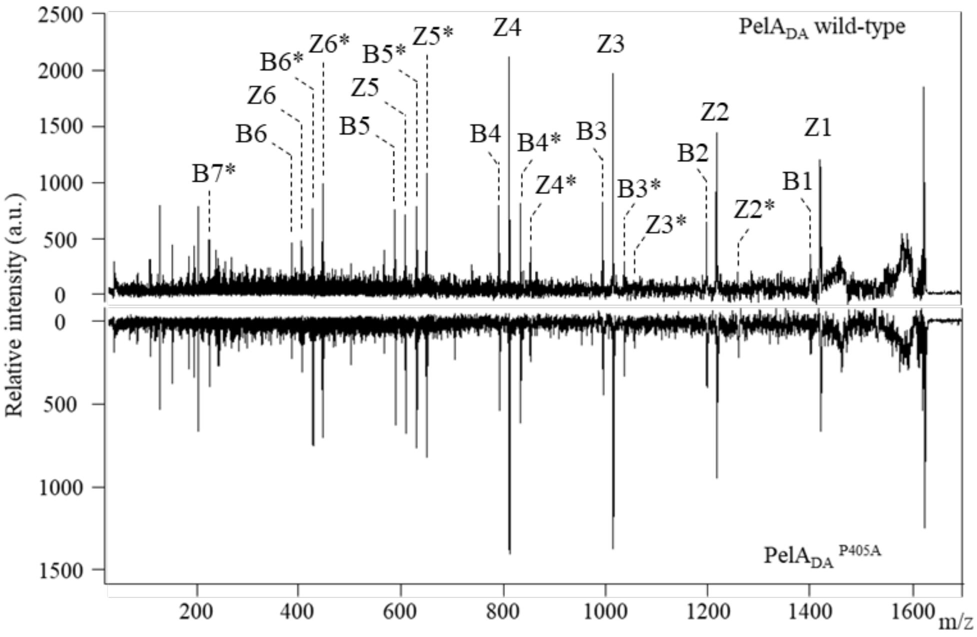

Supplementary Figure 7: MS-MS fragmentation of the ion at m/z 1625.6 corresponding to the mono-deacetylated octamer of GalNAc obtained post-deacetylation with the wild type PelA$_{DA}$ or the P405A mutant. No difference could be observed in the pattern of fragmentation, no conclusive structure could be established due to the heterogeneity of the 1625.6 ion pool. All fragments resulting from a single fragmentation event are labeled according to the nomenclature developed by Domon and Costello (https://doi.org/10.1007/BF01049915). Due to the heterogeneity in deacetylation resulting in multiple ions at m/z 1625.6, fragments could possess or not the deacetylated residue. * was added to the labels if the neutral loss includes the deacetylated residue. All m/z ratio could be found in the supplemental Table S3.

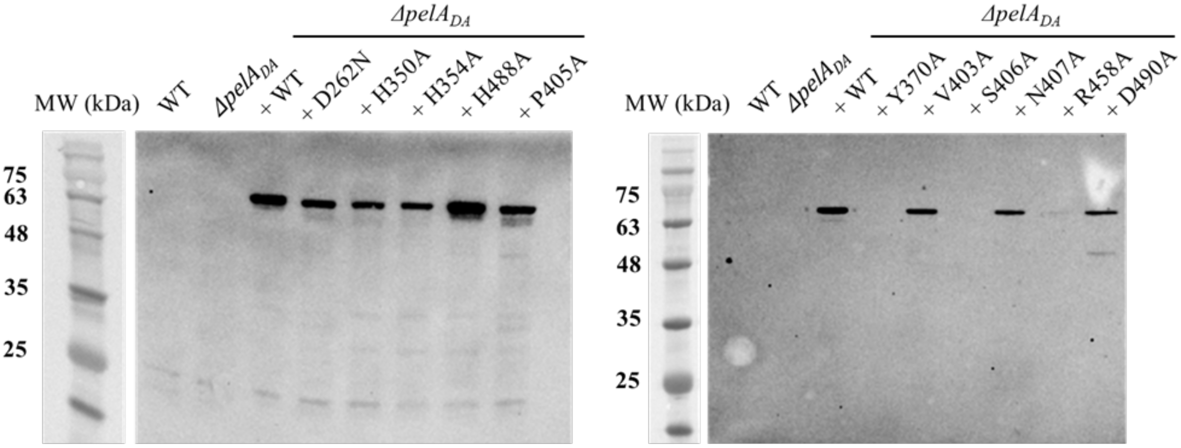

Supplementary Figure 8: *In vivo* expression levels and stability of PelA$_{DA}$ wild-type and mutants. OD$_{600}$ normalized samples of *B. cereus* ATCC 10987 whole cell lysates expressing PelA$_{DA}$ wild-type and mutants were analyzed by Western blotting with a polyclonal PelA$_{DA}$ antibody.

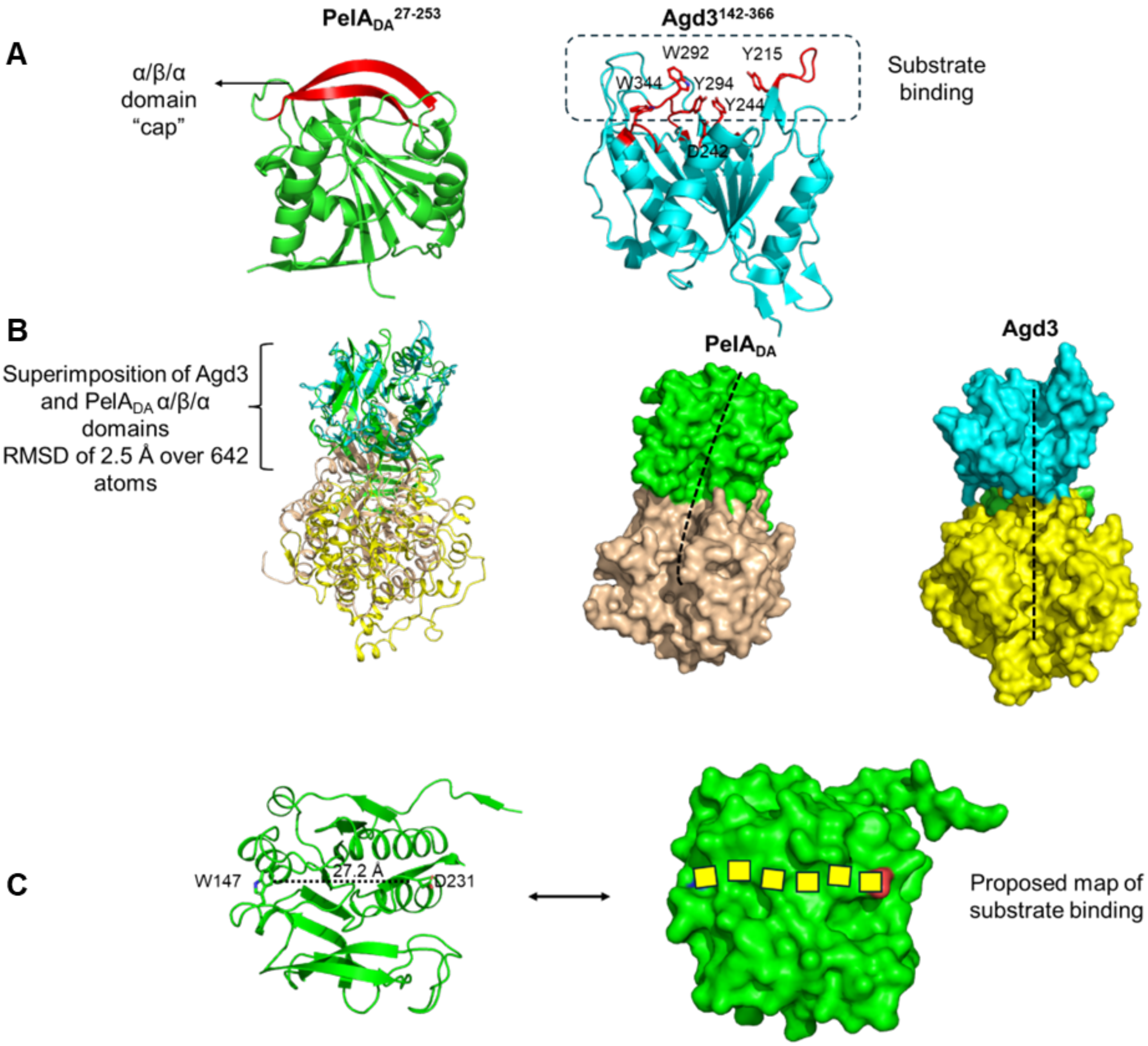

Supplementary Figure 9: PelA$_{DA}$ and Agd3 contain a CBM that is arranged in tandem with the respective CE domains. (A) Cartoon representation depicting the structural differences between the CBMs of PelA$_{DA}$ (left) and Agd3 (right). (B) Superimposition of CBMs of PelA$_{DA}$ and Agd3. Surface views demonstrate the differences in the substrate binding grooves across the two proteins. (C) Distance measurements demonstrating that the CBM binding groove spans 6 subsites. CBM, carbohydrate binding module.

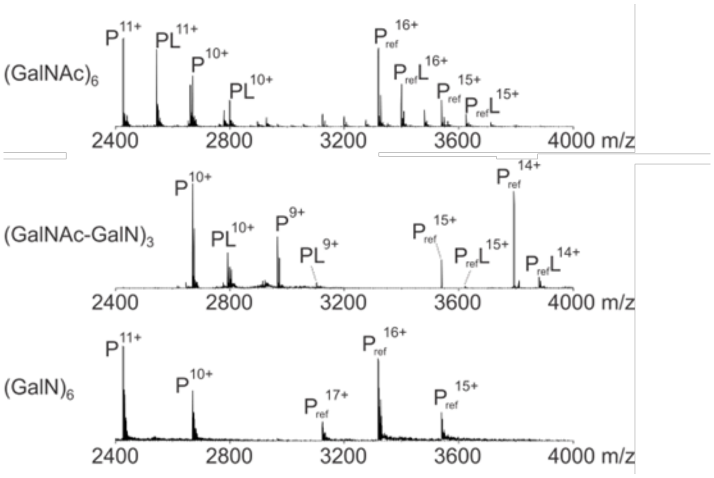

Supplementary Figure 10: Representative ESI mass spectra acquired in positive mode for aqueous ammonium acetate solutions of 4 µM $PelA_{DA}^{27-253}$ wild-type (≡ P) in presence of 200 µM of $(GalNAc)_6$, $(GalNAc\text{-}GalN)_3$ or $(GalN)_6$ ligands (≡ L) and 2 µM streptavidin (P_{ref}). Molecular weight (MW) of $PelA_{DA}^{27-253}$ wild-type was found to be 26,693 Da, MW of P_{ref} was measured to be 53,085 Da. MWs of P complexes with ligands $(GalNAc)_6$, or $(GalNAc\text{-}GalN)_3$ (PL) were measured to be larger than MW of P by 1290.5 and 1224.5 Da, correspondingly. MWs of nonspecific complexes P_{ref} with $(GalNAc)_6$ or $(GalNAc\text{-}GalN)_3$ ($P_{ref}L$) were larger than MWs of P_{ref} by 1290.5 or 1224.5 Da.

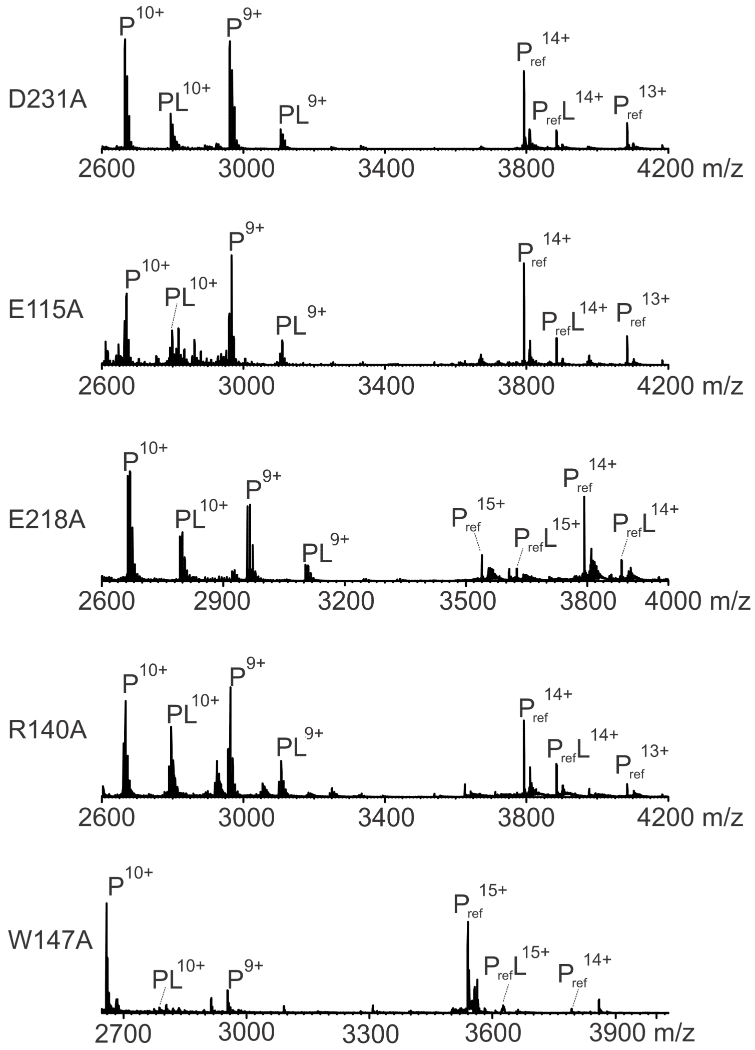

Supplementary Figure 11: Representative ESI mass spectra acquired in positive mode for aqueous ammonium acetate solutions of 8 μM of $PelA_{DA}^{27\text{-}253}$ mutants (≡ P) in the presence of 200 μM of 6-mer of α-1,4-GalNAc ($(GalNAc)_6$ ≡ L) and 2 μM streptavidin (P_{ref}). MWs of $PelA_{DA}^{27\text{-}253}$ mutants were found to be 26,649 Da (D231A), 26,635 Da (E115A and E218A), 26,608 Da (R140A) and 26,578 Da (W147A). Due to binding of the mutants to L, MW of each mutant complex (PL) increased by 1290.5 Da compared to corresponding mutant MW. No specific binding was detected for W147A mutant. MW of P_{ref} was measured to be 53,085 Da, MW of its complex with L was larger by 1290.5 Da.

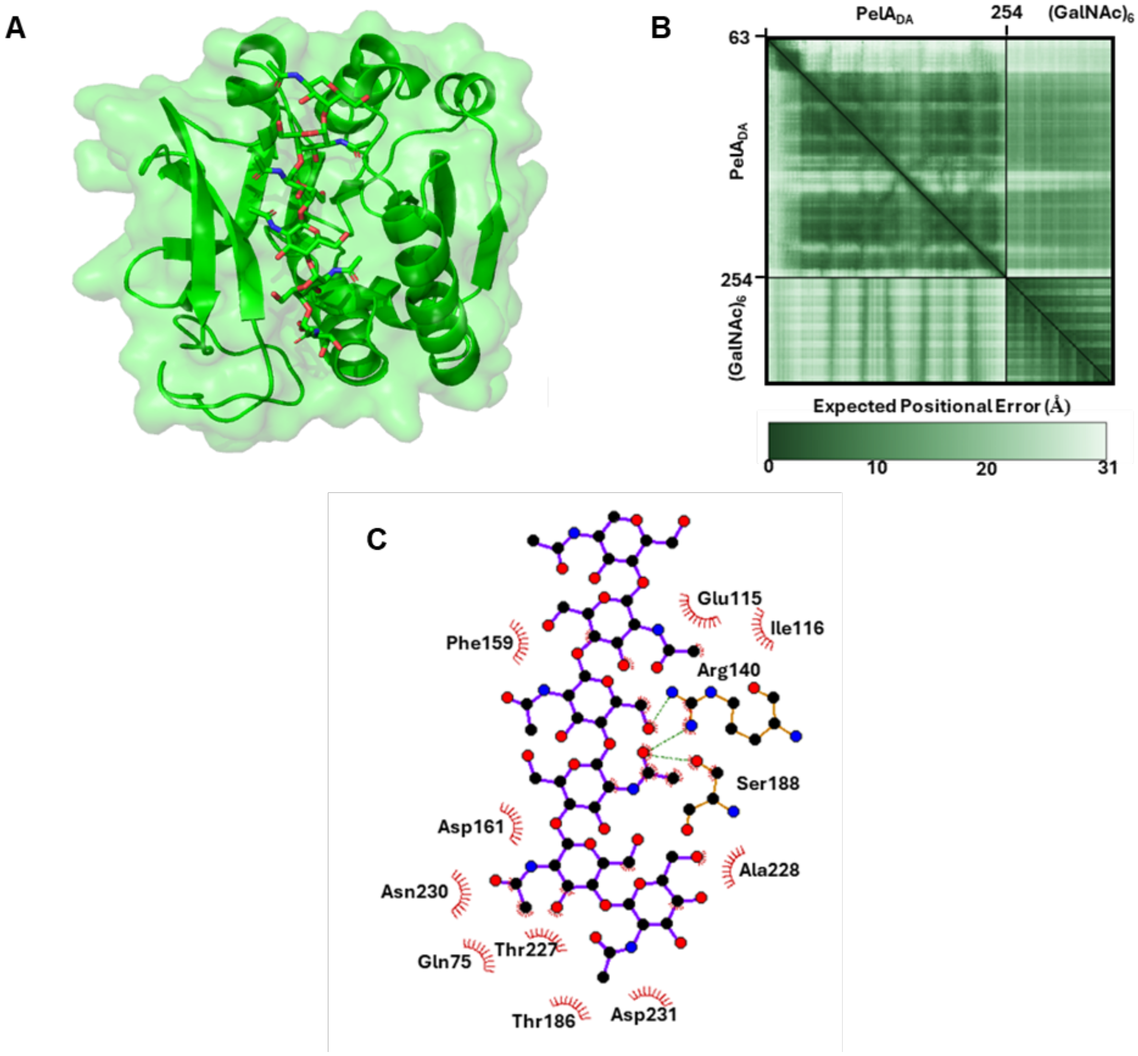

Supplemental Figure 12: AF3 predicted binding of (GalNAc)$_6$ to the CBM domain. (A) Illustration of PelA$_{DA}$ bound to (GalNAc)$_6$ (ipTM = 0.4, pTM = 0.59). PelA$_{DA}$ is depicted in a cartoon representation with a semi-transparent surface. (B) Residue-residue predicted alignment error (PAE) plots for the PelA$_{DA}$-(GalNAc$_6$) complex (4). The values range from 0-31 Å. (C) LIGPLOT diagram showing PelA$_{DA}$-(GalNAc$_6$) interactions. Hydrophobic contacts between residues of PelA$_{DA}$ and (GalNAc)$_6$ are indicated by the brick-red spoked arcs. Hydrogen bonds are depicted as dashed green lines.

References

1. Zhang, G., Wang, W., Deng, A., Sun, Z., Zhang, Y., Liang, Y., Che, Y., and Wen, T. (2012) A Mimicking-of-DNA-Methylation-Patterns Pipeline for Overcoming the Restriction Barrier of Bacteria. *PLoS Genet.* **8**, e1002987

2. Whitfield, G. B., Marmont, L. S., Bundalovic-Torma, C., Razvi, E., Roach, E. J., Khursigara, C. M., Parkinson, J., and Howell, P. L. (2020) Discovery and characterization of a Gram-positive Pel polysaccharide biosynthetic gene cluster. *Plos Pathog*. **16**, e1008281

3. Lee, M. J., Geller, A. M., Bamford, N. C., Liu, H., Gravelat, F. N., Snarr, B. D., Mauff, F. L., Chabot, J., Ralph, B., Ostapska, H., Lehoux, M., Cerone, R. P., Baptista, S. D., Vinogradov, E., Stajich, J. E., Filler, S. G., Howell, P. L., and Sheppard, D. C. (2016) Deacetylation of Fungal Exopolysaccharide Mediates Adhesion and Biofilm Formation. *mBio*. **7**, e00252-16

4. Elfmann, C., and Stülke, J. (2023) PAE viewer: a webserver for the interactive visualization of the predicted aligned error for multimer structure predictions and crosslinks. *Nucleic Acids Res.* **51**, W404–W410
